# Antibacterial and antiviral potential of harmalacidine hydrochloride, a β-carboline alkaloid, against respiratory tract pathogens: *Staphylococcus aureus* and H1N1 influenza virus

**DOI:** 10.1371/journal.pone.0335014

**Published:** 2025-11-04

**Authors:** Manal A. Alossaimi, Fatma M. Abdel Bar, Engy Elekhnawy, Mai H. ElNaggar

**Affiliations:** 1 Department of Pharmaceutical Chemistry, College of Pharmacy, Prince Sattam Bin Abdulaziz University, Al-Kharj, Saudi Arabia; 2 Department of Pharmacognosy, College of Pharmacy, Prince Sattam Bin Abdulaziz University, Al-Kharj, Saudi Arabia; 3 Department of Pharmacognosy, Faculty of Pharmacy, Mansoura University, Mansoura, Egypt; 4 Microbiology and Immunology Department, Faculty of Pharmacy, Tanta University, Tanta, Egypt; 5 Department of Pharmacognosy, Faculty of Pharmacy, Kafrelsheikh University, Kafrelsheikh, Egypt; University of Coimbra: Universidade de Coimbra, PORTUGAL

## Abstract

Respiratory infections remain a leading cause of morbidity and mortality, necessitating new therapeutic strategies. This study evaluated the antiviral and antibacterial activities of harmine, harmaline, and harmalacidine hydrochloride against H1N1 influenza virus and *Staphylococcus aureus*, key respiratory pathogens. The in vitro antiviral activity of the tested compounds against the H1N1 virus was evaluated using a plaque assay. Harmalacidine hydrochloride demonstrated notable activity, with an IC_50_ of 68.2 ± 0.8 µg/mL, while harmine and harmaline showed no significant effects at non-cytotoxic concentrations. The potential antibacterial action of the tested compounds was initially investigated by agar well diffusion method, which revealed clear zones of inhibition around the wells. Subsequently, their minimum inhibitory concentrations (MICs) were recorded using the broth microdilution method. Harmalacidine hydrochloride exhibited the highest antibacterial action with MICs from 16 to 128 µg/mL. Based on these findings, further investigations were conducted to assess the effect of harmalacidine hydrochloride on membrane integrity and permeability, cellular morphology, and biofilm formation. A noticeable reduction (*p* < 0.05) in the membrane integrity and a distinct escalation (*p* < 0.05) in the permeability were noticed in 46.15% and 53.85% of the tested isolates, respectively. Moreover, scanning electron microscopy revealed pronounced distortion in cellular morphology following harmalacidine hydrochloride treatment. The compound also exhibited antibiofilm activity, as demonstrated by the crystal violet assay, alongside a downregulation of biofilm-associated gene expression. Molecular docking revealed that harmalacidinium ion binds strongly to the Accessory Gene Regulator A (*AgrA*) of *S. aureus*, suggesting antibacterial activity through inhibition of quorum sensing-mediated virulence. It also showed high affinity for H1N1 neuraminidase and polymerase basic protein 2 (PB2), indicating potential antiviral activity. However, experimental enzyme assays and *in vivo* studies are required to confirm the proposed antiviral and antibacterial mechanisms.

## Introduction

Respiratory infections are common in all parts of the world, and such infections have received a huge attention in the COVID-19 era. Respiratory infections could be induced by viruses like the influenza virus or by many bacterial species [1,2]. Infections triggered by viruses are regarded as a main cause of morbidity as well as mortality all over the world [3]. The influenza virus is one of the most aggressive viruses that affects a very large number of patients every year. Conventional antiviral drugs are limited by their low availability, variable efficacy, and high cost. Therefore, there is a pressing need to explore alternative antiviral agents [4]. *Staphylococcus aureus* is a common cause of lower respiratory tract infections. This infection is usually associated with various types of ailments in the lungs, including empyema, pleural effusions, abscesses, and necrosis [5].

Influenza virus infection markedly increases the risk and severity of secondary *S. aureus* infection, often associated with higher severity and mortality, particularly during pandemic outbreaks [6–8]. Influenza A virus is reported to trigger host physiological changes that cause *S. aureus* biofilm dispersal, promoting a shift from harmless colonization to invasive infection [8,9]. Influenza virus has also been shown to directly bind to bacterial surfaces, enhancing their adherence to respiratory epithelial cells [10]. Biofilm formation by *S. aureus* bacteria is considered a significant characteristic, which can protect *S. aureus* against different eradication measures and the host defences [11]. In addition to the large panel of virulence factors of *S. aureus* bacteria, they are acquiring resistance to various antibiotics, which is a major obstacle to the treatment of their infections [12].

The complexity and severity of viral and bacterial co-respiratory infection underscore the need for effective treatment approaches that can address both pathogens simultaneously. The use of broad-spectrum treatment could revolutionize infectious disease management by simplifying therapy and reducing the risk of resistance.

β-Carbolines are a class of naturally occurring alkaloids with a distinctive pyrido-indole structure. They are widely distributed in nature and found in various plants and foods [13]. They exhibit diverse pharmacological effects with broad-spectrum efficacy against bacteria, fungi, and viruses, making them strong candidates for development as “magic bullet” drugs [14–16]. β-Carbolines also represent promising candidates for developing new antimicrobial agents and combating antibiotic resistance [17,18]. Harmine and harmol, isolated from *Peganum harmala* L., were reported to exert significant antibacterial activity against *Proteus vulgaris* and *Bacillus subtilis* [19]. While nigritanine, a β-carboline alkaloid isolated from *Strychnos nigritana*, showed potent activity against *S. aureus* [20]. Moreover, several β-carboline alkaloids have been reported to exhibit antiviral activity against a variety of viruses. Acetyl-β-carboline has shown promising antiviral activity against hepatitis B virus (HBV) in cell-based studies, with a favorable safety profile and potential effectiveness against drug-resistant strains [21]. It was suggested to exert its antiviral activity against hepatitis B virus (HBV) primarily by targeting the HBV polymerase enzyme [21]. 1-Formyl-β-carboline derivatives and 9-butyl-harmol were reported to be potent inhibitors of different genotypes of Newcastle disease virus (NDV) as well as several other paramyxoviruses [22,23]. 9-Butyl-harmol inhibited paramyxovirus replication via dual targeting of Glycogen Synthase Kinase-3 (GSK-3β) and Heat Shock Protein 90 (HSP90β) and boosting the host immune response [23]. 1-Formyl-β-carboline derivatives were also reported to inhibit the Newcastle disease virus replication by disrupting its adsorption and entry into host cells [22]. β-Carboline derivatives also demonstrated antiviral activity against poliovirus and herpes simplex virus (HSV-1) [24–27]. Harmol and 9-*N*-methylharmine were reported to selectively suppress the replication of dengue virus in cell cultures without exhibiting any direct virucidal activity [28]. The β-carboline moiety containing manzamine alkaloids exhibited significant activity against HIV-1, opportunistic infections associated with AIDS [29]. β-Carboline derivatives were also reported to possess antiviral activity against influenza viruses [30,31].

The broad-spectrum antiviral and antibacterial activity of β-carboline derivatives prompted us to investigate the potential of the major β-carboline alkaloids derived from *P. harmala* seeds against respiratory tract infections caused by the influenza virus and *S. aureus* bacteria.

## Materials and methods

### Source of compounds

The compounds used in this study included harmalacidine hydrochloride, previously isolated by our research group from the *n*-butanol fraction of *Peganum harmala* L. seed extract [32]. The purity of isolated harmalacidine hydrochloride was confirmed by the NMR spectral data (S2 Table and S1–S6 Figs). Commercially available alkaloids, including harmine obtained from Biosynth Ltd. (Compton, Berkshire, UK), and harmaline purchased from Merck KGaA (Darmstadt, Germany) were also used in the study.

### Antiviral action

#### Viruses, chemicals, and cells.

For propagation of the H1N1 virus, Vero-E6 cells were utilised in Dulbecco’s modiﬁed Eagle’s medium (DMEM) with added substances, including 10% fetal bovine serum (FBS) and 1% penicillin/streptomycin mixture. The cells were incubated at 37 °C in the presence of 5% CO_2,_ then the supernatant was centrifuged to eliminate the cell debris, and it was subjected to the plaque assay. All the utilised chemicals and media were purchased from Merck, UK.

#### MTT test.

The main purpose of this test was to detect the half-maximal cytotoxic concentration (CC_50_) of the tested compounds according to the previously explained method [33,34]. Briefly, the cells were seeded in 96-well plates and incubated for 24 h at 37 °C in 5% CO_2_, and after 24 hours, the cells were treated with various concentrations of the tested compounds. The supernatant was then discarded after 24 hours, and cell monolayers were washed with sterile phosphate-buffered saline (PBS) three times. The MTT solution (20 µL of 5 mg/mL stock solution) was added to each well and incubated at 37 °C for four hours, followed by medium aspiration. In each well, the formed formazan crystals were dissolved with 100 µL of dimethyl sulfoxide (DMSO). The absorbance (Ab) of the formed formazan was recorded by an ELISA reader (Sunrise Tecan, Switzerland) at 540 nm. The percentage of cell viability was calculated by the formula:


$$The\;percentage\;of\;the\;cell\;viability=\frac{Absorbance\;of\;the\;cells\;with\;treatment}{Absorbance\;of\;the\;cells\;without\;treatment}\times 100$$

After that, a curve was constructed for the viability percentage and the compound concentrations.

#### Plaque assay.

It was performed according to the previously reported method on Vero-E6 cells to investigate the potential antiviral activity of the tested compounds in six-well plates [33,35]. After the propagation of the H1N1 virus, it was 10-fold serially diluted in DMEM. Then, the viruses were put onto the Vero-E6 cells, and they were incubated at 37 °C for 60 min. After that, the suspension of the viruses was taken off, and DMEM, with 2% agarose containing the tested compounds at safe concentrations (at concentrations below the previously determined CC_50_ to ensure minimal cytotoxicity), was overlaid onto Vero-E6 cells. For each compound, a full dose-response curve was generated using five serial dilutions, covering a range from sub-therapeutic to near-CC_50_ levels. The agarose was then left to solidify, and the plates were incubated for 72 h at 37 °C in 5% CO_2_. Then, 10% formalin was added for fixation purposes for one hour. Finally, the plaque count was recorded, and the percentage of reduction of the plaque formation was determined:


$$\%\;\text{inhibition}=\frac{\text{Untreated\textbackslash{} viral\textbackslash{} count}-\text{treated\textbackslash{} viral\textbackslash{} count}}{\text{untreated\textbackslash{} viral\textbackslash{} count}}\;\times 100$$

### Antibacterial activity

#### Agar well diffusion method and determination of the minimum inhibitory concentration (MIC).

The tested bacterial isolates were thirteen *S. aureus* clinical isolates (methicillin-sensitive isolates) from the microbiology and immunology department, faculty of pharmacy, Tanta University. The isolates were obtained from different specimens from Tanta University Hospital (wound, sputum, and blood). A loopful of the bacterial suspensions was inoculated onto the surface of Muller-Hinton agar plates. Wells were ten-formed, and they were filled with 2000 μg/mL of each tested compound, and the plates were incubated for 18 h at 37°C. Inhibition zones were inspected, indicating antibacterial activity [36]. A positive control (vancomycin) and a negative control (DMSO) were used. The MICs were determined using the broth microdilution technique [37]. In brief, two-fold serial dilutions of the tested compounds, starting from 2048 μg/mL to 1 μg/mL, were added to each well with the bacterial suspension. After incubation, the MIC values were recorded as the lowest concentrations that inhibited bacterial growth.

#### Impact on the bacterial membrane integrity and permeability.

The impact of the tested compound on the integrity of the *S. aureus* membrane was revealed via determination of the absorbance (Ab) at 260 nm by UV-Vis spectrophotometer (SHIMADZU, Japan) according to the previously reported method [38].

The impact of the tested compound on *S. aureus* membrane permeability was inspected via tracing the liberation of β-galactosidase enzyme from the cell interior to outside by *O*-nitrophenyl-β-galactopyranoside (ONPG), which is considered a substrate for the enzyme. The formed *O*-nitrophenol (ONP) was detected at 420 nm by an ELISA reader [39].

#### Scanning electron microscope (SEM).

The impact of the tested compound on the morphology of *S. aureus* was elucidated by examination with SEM (Hitachi, Japan). The bacterial isolates before and after treatment were visualised using SEM after fixation using ethanol as previously reported [40].

### Antibiofilm potential

#### Crystal violet assay.

*S. aureus* isolates were classified into non-forming, weak, moderate, and strong forming according to their ability to form biofilm by crystal violet assay in a 96-well plate [36]. After allowing the bacterial isolates to form biofilm in the microtitration plates, crystal violet solution was utilised for staining the biofilm-forming cells as previously described by measuring Ab at 490 nm [36].

#### qRT-PCR.

In the current investigation, qRT‐PCR was employed for molecular study of the impact of the tested compound on the biofilm formation by *S. aureus*. After the extraction of the total RNA using a kit from Qiagen, Germany, they were converted into cDNA using a cDNA synthesis kit (Promega, USA). The concentration and purity of the total RNA and cDNA were assessed using a Nanodrop spectrophotometer. Then, qRT-PCR was run using primers listed in Table 1 using *16S rRNA* as a reference gene. Relative gene expression was calculated using the 2^−ΔΔCt^ method [41].

**Table 1:** The utilised primer sequences.

Primer	Sequence (5’ to 3’)
*16S rRNA*	F: GGGACCCGCACAAGCGGTGG R: GGGTTGCGCTCGTTGCGGGA
*icaA*	F: GAGGTAAAGCCAACGCACTC R: CCTGTAACCGCACCAAGTTT
*fnbA*	F: AAATTGGGAGCAGCATCAGT R: GCAGCTGAATTCCCATTTTC
*cna*	F: AATAGAGGCGCCACGACCGT R: GTGCCTTCCCAAACCTTTTGAGC

### Statistical analysis

Assays were performed in triplicate, and the obtained data were represented as mean ± standard deviation (SD). Comparisons between different groups were conducted by ANOVA followed by a post-hoc test. The level of significance was at *p* < 0.05. The statistics were processed by GraphPad Software 8 (San Diego, CA).

### Molecular docking study

AutoDock Vina 1.2.6 [42,43] was used to carry out the molecular docking study utilizing protein structure with PDB codes presented in S1 Table, following the protocol described in the Supporting Information (S1 Method).

## Results

### Antiviral potential

#### Cytotoxicity of the tested compounds.

**Fig 1:**
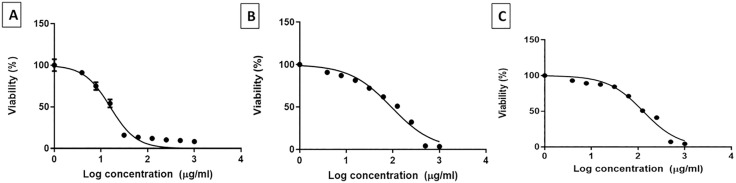
A curve showing the cytotoxicity of A) harmine (CC_50_ of 20.8 ± 1.1 µg/mL), B) harmaline (CC_50_ of 130.2 ± 1.2 µg/mL), and C) harmalacidine hydrochloride (CC_50_ of 141.7 ± 0.9 µg/mL) on Vero-E6 cells.

The cytotoxic effects of harmine, harmaline, and harmalacidine hydrochloride were evaluated on Vero-E6 cells using the MTT assay. The 50% cytotoxic concentration (CC_50_) values were determined from dose-response curves (Fig 1). Harmine exhibited the highest cytotoxicity with a CC_50_ of 20.8 ± 1.1 µg/mL. In contrast, harmaline and harmalacidine hydrochloride showed considerably lower cytotoxicity, with CC_50_ values of 130.2 ± 1.2 µg/mL and 141.7 ± 0.9 µg/mL, respectively. These results indicated a broader safety margin for harmaline and harmalacidine hydrochloride compared to harmine, supporting their potential for further biological evaluation.

#### Antiviral activity of the tested compounds.

**Fig 2:**
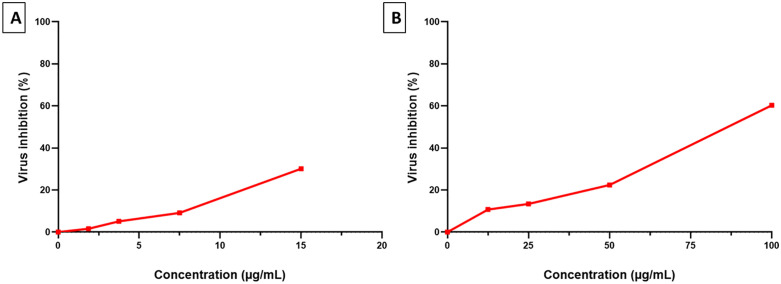
Antiviral activity of A) harmine (IC_50_ was not determined as it would be above the safe concentration) and B) harmalacidine hydrochloride (IC_50_ of 68.2 ± 0.8 µg/mL) on H1N1 influenza virus.

The antiviral potential of the tested compounds was tested against the H1N1 influenza virus, and their values of the half maximal inhibitory concentration (IC_50_) were recorded (Fig 2).

### Antibacterial potential

#### Agar well diffusion and MICs.

The three tested compounds presented antibacterial action on *S. aureus* by revealing inhibition zones around the wells that contained them in the agar diffusion test. Thus, the MIC values were recorded by the broth microdilution method. The MIC values of harmine, harmaline, and harmalacidine hydrochloride ranged from 512 to 1024, from 256 to 1024, and from 16 to 128 µg/mL, respectively (Table 2).

**Table 2:** The recorded MIC values of harmine, harmaline, and harmalacidine hydrochloride.

Isolate	MIC values (µg/mL)		
	Harmine	Harmaline	Harmalacidine hydrochloride
S1	1024	256	16
S2	1024	512	16
S3	1024	512	32
S4	1024	512	16
S5	512	512	32
S6	512	1024	32
S7	1024	1024	64
S8	1024	1024	64
S9	1024	256	16
S10	1024	256	32
S11	512	1024	128
S12	512	512	128
S13	1024	1024	32

#### Membrane integrity and permeability.

**Fig 3:**
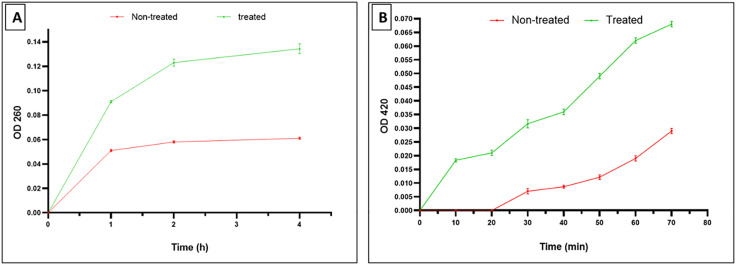
Charts revealing A) the significant decrease (*p* < 0.05) in the membrane integrity and B) the significant increase (*p* < 0.05) in the membrane permeability after treatment with harmalacidine hydrochloride in a representative *S. aureus* isolate (n = 3 as technical repeats of the same isolate).

The effect of harmalacidine hydrochloride on the membrane integrity of *S. aureus* isolates was illuminated by exploring the discharge of the materials that possess absorbance at 260 nm, from the interior of the bacterial cells. We observed a noticeable reduction (*p* < 0.05) in the membrane integrity in 46.15% of *S. aureus* isolates (Fig 3A). In addition, a distinct escalation (*p* < 0.05) in the permeability of *S. aureus* membrane was found after treatment with harmalacidine hydrochloride in 53.85% of the isolates (Fig 3B).

#### SEM.

**Fig 4:**
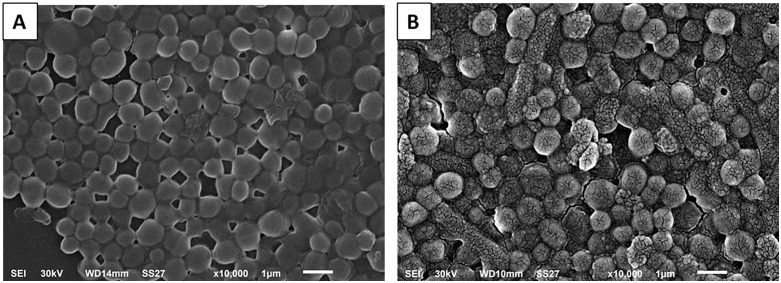
Scanning electron micrograph of a biofilm forming *S. aureus* isolate A) before and B) after treatment with harmalacidine hydrochloride.

The influence of harmalacidine hydrochloride on the morphology of *S. aureus* isolates was studied by SEM (Fig 4). As noticed, the bacterial surfaces were rough, irregular, and lysed in certain cells after treatment with harmalacidine hydrochloride.

### Antibiofilm action

#### Crystal violet assay.

The influence of harmalacidine hydrochloride on the ability to form biofilm by *S. aureus* isolates was illuminated by the crystal violet method. It was noticed that harmalacidine hydrochloride has diminished the percentage of *S. aureus* isolates that strongly and moderately form biofilm from 53.85% to 7.69% (Table 3). Chi-square test was conducted, and we have found that the shift from the strongly and moderately forming biofilm isolates to non and weakly forming biofilm isolates was significant (*p* > 0.05).

**Table 3:** Impact of harmalacidine hydrochloride on the biofilm formation of **S. aureus*.*

Biofilm formation	Number of isolates	
	Before treatment	After treatment
Non-forming	2	5
Weak	4	7
Moderate	5	1
Strong	2	0

#### Biofilm gene expression.

**Fig 5:**
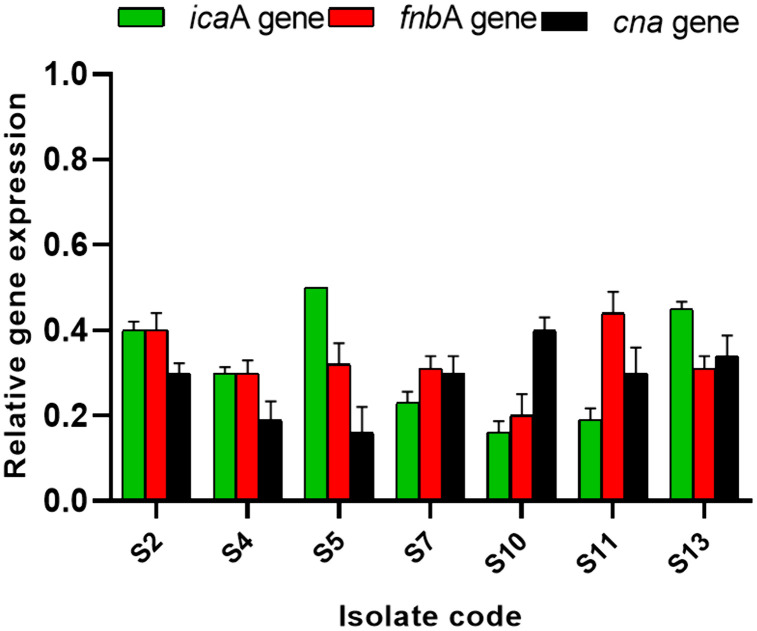
The downregulating effect of harmalacidine hydrochloride on the biofilm genes in seven *S. aureus* isolates.

The influence of harmalacidine hydrochloride on the gene expression of the biofilm genes in *S. aureus* isolates was illuminated using qRT-PCR. The studied genes were the *ica*A gene (intercellular adhesion gene), the *fnb*A gene (fibronectin-binding protein A), and the *cna* gene (collagen-binding protein). Remarkably, harmalacidine hydrochloride revealed a downregulating impact on the expression of the tested genes in 53.85% of *S. aureus* isolates (Fig 5).

## Discussion

In the current COVID-19 era, we have a worldwide dissemination of respiratory tract illnesses [44]. So, it is crucial to disclose different options for the management of such ailments. It is known that plants are very rich sources of various bioactive phytochemicals that could provide us with solutions for the treatment of different diseases [45]. Many researchers have investigated the potential therapeutic action of many bioactive compounds from plants [46–49]. Herein, we examined the antiviral and antibacterial action of harmine, harmaline, and harmalacidine hydrochloride against the H1N1 influenza virus and *S. aureus* bacteria. Both pathogens are very common causative agents for many respiratory tract infections [50].

The annual estimate of the infections caused by the influenza virus is 20–30% in children and 5–10% in adults all over the world [51]. Such viral infections are frequently linked to lower respiratory tract infections with relatively great rates of hospitalization [52]. In addition to this, there is an increase in the percentage of drug-resistant influenza strains [53]. Therefore, it is critical to screen for novel anti-influenza therapeutics that have antiviral action with low toxicity.

The *in vitro* antiviral action of harmine, harmaline, and harmalacidine hydrochloride was explored against the influenza virus (H1N1) using the plaque assay. Such an assay is frequently utilised for counting infectious viruses by determining the number of plaques they produce in the infected cells [54]. Here, harmalacidine hydrochloride revealed an IC_50_ value of 68.2 ± 0.8 µg/mL on the H1N1 influenza virus. The value of IC_50_ denotes the necessary concentration of the drug that can produce an *in vitro* inhibition of 50% of the viral particles [55]. Certain studies have documented the anti-influenza action of certain phytochemicals from plants [30,56–58].

The current increasing misuse of antibiotics has resulted in the dissemination of resistance among many bacterial species. Consequently, there is a high need for alternative treatments [59]. Herein, the antibacterial action of harmine, harmaline, and harmalacidine hydrochloride on *S. aureus* isolates was explored. Harmine, harmaline, and harmalacidine hydrochloride had MIC values that ranged from 512 to 1024, from 256 to 1024, and from 16 to 128 µg/mL, respectively.

Many antibacterials target the bacterial membranes to elicit their action [60]. In this study, the effect of harmalacidine hydrochloride was investigated on the membrane integrity and permeability. This compound was further studied as it revealed the best antibacterial action on *S. aureus* isolates with the lowest MIC values. Harmalacidine hydrochloride exhibited a marked reduction in the integrity of 46.15% of *S. aureus* isolates. Membrane integrity is regarded as an important indication of the bacterial membrane quality. If there is a destructive effect on the bacterial membrane, its integrity would decrease [61]. This in turn would lead to increasing the discharge of the nucleic acids, which absorb at 260 nm, from the cell to outside [62]. Also, there is an additional property of the bacterial membrane, and it is the membrane permeability. In the current investigation, the impact of harmalacidine hydrochloride on the membrane permeability was inspected. Prominently, harmalacidine hydrochloride has raised the membrane permeability in 53.85% of *S. aureus* isolates. The bacterial membranes aid in the conservation of the homeostasis of the cells and subsequently protect the various physiological cellular processes [63,64].

The major focus of different research is on developing new antimicrobials; however, the antivirulence approaches are currently drawing major attention [65]. Thus, we investigated the antibiofilm action of harmalacidine hydrochloride against *S. aureus*. Using the crystal violet assay, we found that harmalacidine hydrochloride has diminished the percentage of *S. aureus* isolates strongly and moderately forming biofilm from 53.85% to 7.69%. Also, qRT-PCR revealed that harmalacidine hydrochloride has downregulated the biofilm genes in 53.85% of *S. aureus* isolates. This is an important finding that supports the antibacterial action of harmalacidine hydrochloride as certain antibiotics could have a negative effect on the bacterial virulence along with their growth inhibitory action. This strongly potentiates their antibacterial activity. For example, previous studies have documented the inhibitory action of linezolid on the production of the virulence factors by *S. aureus* [66,67]. Antimicrobial compounds are usually more effective when they also have antibiofilm activity.

We acknowledge that the absence of *in vivo* validation of the tested compounds is a limitation in our study. While the *in vitro* results have demonstrated promising antiviral and antibacterial actions and provide initial mechanistic insights, the efficacy and safety of the tested compounds in animal models remain to be established. Future studies are planned to evaluate these compounds in appropriate *in vivo* models to confirm their therapeutic efficacy.

Molecular docking is a powerful computational technique that predicts the interaction between ligand(s) and a target protein(s), providing valuable insights into their mechanism of action. A docking study was performed to explore the binding affinity, orientation, and potential inhibitory effects of the tested harmala alkaloids on potential targets of *S. aureus* and H1N1. The reported anti-quorum-sensing activity of harmine against *Pseudomonas aeruginosa* and its studied interactions against transcriptional regulators as *LasR* [68] motivated us to investigate the activity of the tested harmala alkaloids against quorum sensing regulators of *S. aureus*. The Accessory Gene Regulator A (*AgrA*) is the master virulence regulator in the quorum sensing system of *S. aureus*, a mechanism that enables the bacterial population to coordinate gene expression in response to cell density. It is a DNA-binding response regulator protein (transcriptional activator) that directly regulates the expression of several genes that are involved in the pathogenicity of *S. aureus*. Targeting *AgrA* for inhibition disrupts this communication system, effectively impairing the bacteria’s ability to establish infections, evade immune responses, and resist antibiotics. By inhibiting *AgrA*, it is possible to attenuate the virulence of *S. aureus* without exerting selective pressure for resistance, offering a promising strategy for the development of anti-virulence therapies [69,70].

**Fig 6:**
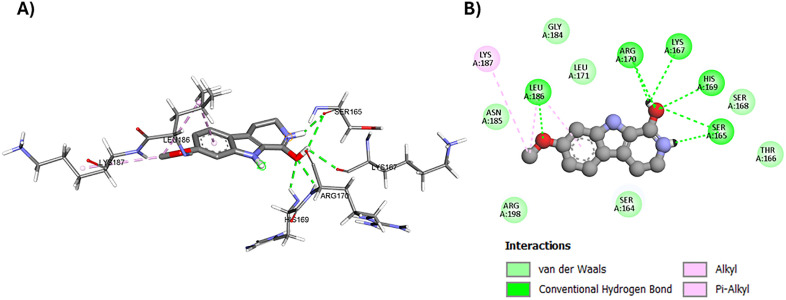
Molecular binding of harmalacidinium ion to the LytTR Domain of the response regulator in the *Staphylococcus aureus* (*AgrA*, PDB code: 3BS1); A) 3D molecular binding model, and B) 2D molecular binding model demonstrating amino acid residues involved in the interaction.

The three harmala alkaloids were docked into the LytTR DNA-binding domain of *AgrA* with harmalacidinium ion showing the highest docking score of −6.569 kcal/mol (Table 4). Harmalacidinium ion showed hydrogen bonding with His-169 amino acid residue that is involved in the enzyme binding to DNA [71,72]. It also showed hydrogen bonding with Ser-165, Lys-167, Arg-170, and Leu-186 amino acid residues and π-alkyl staking with Lys-187 present in the LytTR DNA-binding domain of *AgrA* (Fig 6). Thus, harmalacidine would likely block *AgrA* binding to target DNA promoters and inhibiting virulence factors development.

**Table 4:** Docking scores of the tested Harmala alkaloids against the transcriptional activator receptor (*AgrA*) of *S. aureus*, neuraminidase and polymerase basic protein 2 (PB2) enzymes of H1N1 using AutoDock Vina.

Compound	*AgrA*	Neuraminidase	polymerase basic protein 2 (PB2)
Harmine	−5.567	−6.562	−7.053
Harmaline	−5.444	−6.438	−6.956
Harmalacidinium ion	−6.569	−7.171	−7.215
Coligand	–	−8.507	−10.519

The harmalacidinium ion was also docked against the transcriptional activator *LasR* of *P. aeruginosa* to compare its binding profile with the previously reported alkaloid harmine (data not shown). Its high docking score suggests a potential anti-quorum-sensing effect against *P. aeruginosa*, warranting validation in future experimental studies.

**Fig 7:**
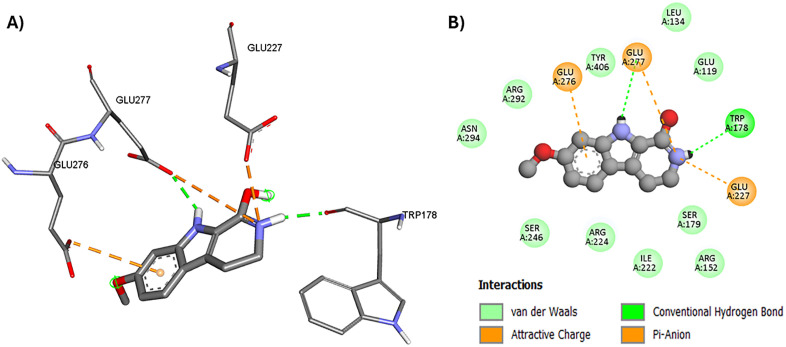
Molecular binding of harmalacidinium ion to H1N1 neuraminidase enzyme (NA, PDB code: 3B7E); A) 3D molecular binding model, and B) 2D molecular binding model demonstrating amino acid residues involved in the interaction.

Neuraminidase (NA) plays a crucial role in the life cycle of the H1N1 influenza virus, primarily by facilitating the release of newly formed viral particles from infected cells. It cleaves sialic acid residues on the host cell surface and viral glycoproteins, preventing newly formed virions from aggregating and allowing efficient viral spread. Harmalacidine hydrochloride showed the highest docking score of the tested harmala alkaloids against H1N1-NA. It showed a reasonable docking score that is close to the co-crystallized ligand, zanamivir (Table 3). The harmalacidinium ion showed H-bonding with Trp-178 amino acid that is involved in the interaction of the neuraminidase (NA) with the co-crystalized inhibitor, zanamivir [73]. In addition, the protonated nitrogen of its piperidine ring formed π-anion bonds with Glu-227, and Glu-277 amino acid residues in the active site of NA (Fig 7). Moreover, the aromatic ring of the harmalacidinium ion exhibited an electrostatic attraction with Glu-276 amino acid residue (Fig 7).

**Fig 8:**
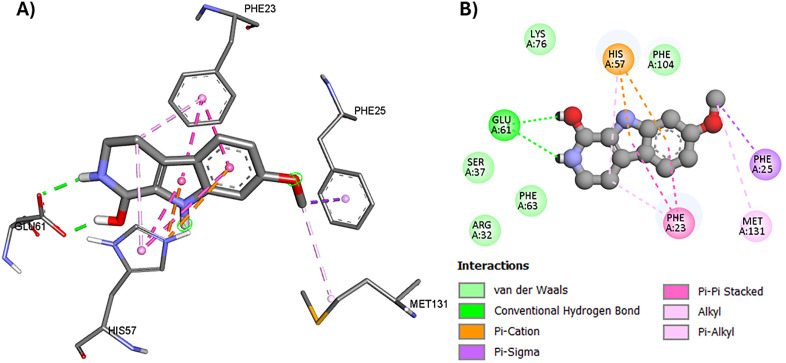
Molecular binding of the harmalacidinium ion to polymerase enzyme basic protein 2 (PB2, PDB code: 4P1U); A) 3D molecular binding model, and B) 2D molecular binding model demonstrating amino acid residues involved in the interaction.

The RNA-dependent RNA polymerase (RdRP) of the influenza virus plays a crucial role in the replication and transcription of its genome, making it an attractive target for antiviral interventions and the development of antiflu therapeutics. Inhibiting this enzyme can significantly hinder the virus’s capacity to multiply [74]. Since β-carboline derivatives have been reported to exhibit antiviral activity against hepatitis B virus (HBV) by targeting the viral polymerase enzyme [21], this enzyme was selected for molecular docking to assess its potential involvement in the observed antiviral activity of harmalacidine hydrochloride. RdRP is a heterotrimeric complex composed of the polymerase acidic protein (PA) and the polymerase basic proteins 1 (PB1) and 2 (PB2), with the assembly of these three subunits being essential for its activity [74]. The investigated *Peganum harmala* alkaloids were evaluated for their interaction with the PB2 subunit of the influenza virus using a molecular docking study. The harmalacidinium ion exhibited the highest docking score among the tested harmala alkaloids (−7.215 kcal/mol, Table 3), indicating moderate inhibitory potential compared to the co-crystallized azaindole inhibitor, which showed a stronger binding affinity (−10.519 kcal/mol). Surprisingly, the harmalacidinium ion showed a binding mode that is very close to that of the co-crystallized ligand, which could be attributed to the shared indole moiety in both structures. Similar to the azaindole cocrystallized inhibitor, the harmalacidinium ion formed an H-bond with Glu-61 [75]. It was also sandwiched between the aromatic side chains of His-57 and Phe-23, forming π-cation, and π-π interactions with them. Moreover, it showed π-sigma, and π-alkyl interactions with Phe-25 and Met-131 respectively (Fig 8).

The docking results presented in this study provide preliminary insights into the potential mechanism of action of harmalacidine hydrochloride and may help explain its higher *in vitro* antimicrobial activity compared with the other investigated compounds against *S. aureus* and H1N1. Nevertheless, further validation using enzyme assays against the tested protein targets is required to substantiate these mechanistic claims.

## Conclusions

Harmalacidine hydrochloride, a β-carboline alkaloid from *P. harmala*, exhibited dual antimicrobial activity against *S. aureus* and H1N1 influenza virus, which are two significant respiratory pathogens. It disrupted the membrane integrity of *S. aureus*, altered cell morphology, and downregulated the expression of biofilm-associated genes, indicating attenuation of bacterial virulence. Molecular docking further suggested interactions of this compound with key viral and bacterial targets, including influenza neuraminidase, polymerase PB2, and the LytTR domain of *S. aureus AgrA*, the master regulator of quorum-sensing-mediated virulence. These findings suggest harmalacidine hydrochloride as a potential therapeutic candidate to simultaneously target viral and bacterial co-infections of the respiratory tract. Broad-spectrum agents of this type could be especially useful during pandemics when vaccines are unavailable or not yet distributed. However, further *in vivo* studies and clinical validation are required to fully verify its effectiveness and safety.

## Supporting information

S1 Table PDB codes of the crystal structures and grid box coordinates for the enzymes used in the docking study. (PDF)

S2 Table ^1^H and ^13^C NMR data of harmalacidine hydrochloride isolated from *Peganum harmala* L. seeds, at 125 and 500 MHz, respectively in CD_3_OD. (PDF)

S1 Fig ^1^H NMR spectrum of harmalacidine hydrochloride (CD_3_OD, 500 MHz). (PDF)

S2 Fig ^13^C NMR spectrum of harmalacidine hydrochloride (CD_3_OD, 125 MHz). (PDF)

S3 Fig DEPT135 spectrum of harmalacidine hydrochloride (CD_3_OD, 125 MHz). (PDF)

S4 Fig COSY spectrum of harmalacidine hydrochloride. (PDF)

S5 Fig HSQC spectrum of harmalacidine hydrochloride. (PDF)

S6 Fig HMBC spectrum of harmalacidine hydrochloride. (PDF)

S1 Raw Data Raw data of Fig 1. (PDF)

S2 Raw Data Raw data of Fig 2A. (PDF)

S3 Raw Data Raw data of Fig 2B. (PDF)

S4 Raw Data Raw data of Fig 3A. (PDF)

S5 Raw Data Raw data of Fig 3B. (PDF)

S6 Raw Data Raw data of Fig 5. (PDF)

S1 Method Molecular docking study. (PDF)
