# Supplementary material for: Antibacterial and antiviral potential of harmalacidine hydrochloride, a β-carboline alkaloid, against respiratory tract pathogens: Staphylococcus aureus and H1N1 influenza virus
Source: PLoS One. 2025 Nov 4;20(11):e0335014. doi: 10.1371/journal.pone.0335014 (PMC12585031; doi:10.1371/journal.pone.0335014)
Supplement: S1 Table — (PDF) [file pone.0335014.s001.pdf]

S1 Table. PDB codes of the crystal structures and grid box coordinates for the enzymes used in the docking study.

Protein	PDB codes	grid box with dimensions (points)	Grid box X, Y and Z coordinates	Reference inhibitor
**Transcriptional activator receptor of *Staphylococcus aureus* (*AgrA*)**	3BS1	90 × 90 × 90	Blind docking 6.27, 9.641, 5.006	-
**H1N1 Neuraminidase**	3B7E	30 × 30 × 30	-29.421, 12.733, and -21.236	Zanamivir [1]
**RNA-dependent RNA polymerase (RdRP)**	4P1U	30 × 30 × 30	-49.069, -6.066, and 3.129	Azaindole inhibitor [2]

References

1. Xu X, Zhu X, Dwek RA, Stevens J, Wilson IA. Structural characterization of the 1918 influenza virus H1N1 neuraminidase. J Virol. 2008;82(21):10493-501. Epub 2008/08/22. https://doi.org/10.1128/jvi.00959-08. PMID: 18715929
2. Clark MP, Ledeboer MW, Davies I, Byrn RA, Jones SM, Perola E, et al. Discovery of a Novel, First-in-Class, Orally Bioavailable Azaindole Inhibitor (VX-787) of Influenza PB2. J Med Chem. 2014;57(15):6668-78. https://doi.org/10.1021/jm5007275. PMID: 25019388
