# Supplementary material for: Antibacterial and antiviral potential of harmalacidine hydrochloride, a β-carboline alkaloid, against respiratory tract pathogens: Staphylococcus aureus and H1N1 influenza virus
Source: PLoS One. 2025 Nov 4;20(11):e0335014. doi: 10.1371/journal.pone.0335014 (PMC12585031; doi:10.1371/journal.pone.0335014)
Supplement: S2 Table — (PDF) [file pone.0335014.s002.pdf]

S2 Table. ^{1}H and ^{13}C NMR data of harmalacidine hydrochloride isolated from *Peganum harmala* L. seeds, at 125 and 500 MHz, respectively in CD_3OD.

**C/H position**	DEPT135	**^{1}H, *J* (Hz)**	**^{13}C**
**1**			166.75
**3**	CH_2	3.89, t (8.9)	43.97
**4**	CH_2	3.20, t (8.8)	20.87
**5**	CH	7.59, d (9.0)	124.33
**6**	CH	6.85, dd (9.0, 2.1)	116.54
**7**			163.90
**8**	CH	6.92, d (1.9)	94.86
**10**			127.54
**11**			127.29
**12**			120.97
**13**			145.43
**OCH_3**	CH_3	3.90, s	56.59
