# Supplementary figures and images for: Antibacterial and antiviral potential of harmalacidine hydrochloride, a β-carboline alkaloid, against respiratory tract pathogens: Staphylococcus aureus and H1N1 influenza virus
Source: PLoS One. 2025 Nov 4;20(11):e0335014. doi: 10.1371/journal.pone.0335014 (PMC12585031; doi:10.1371/journal.pone.0335014)

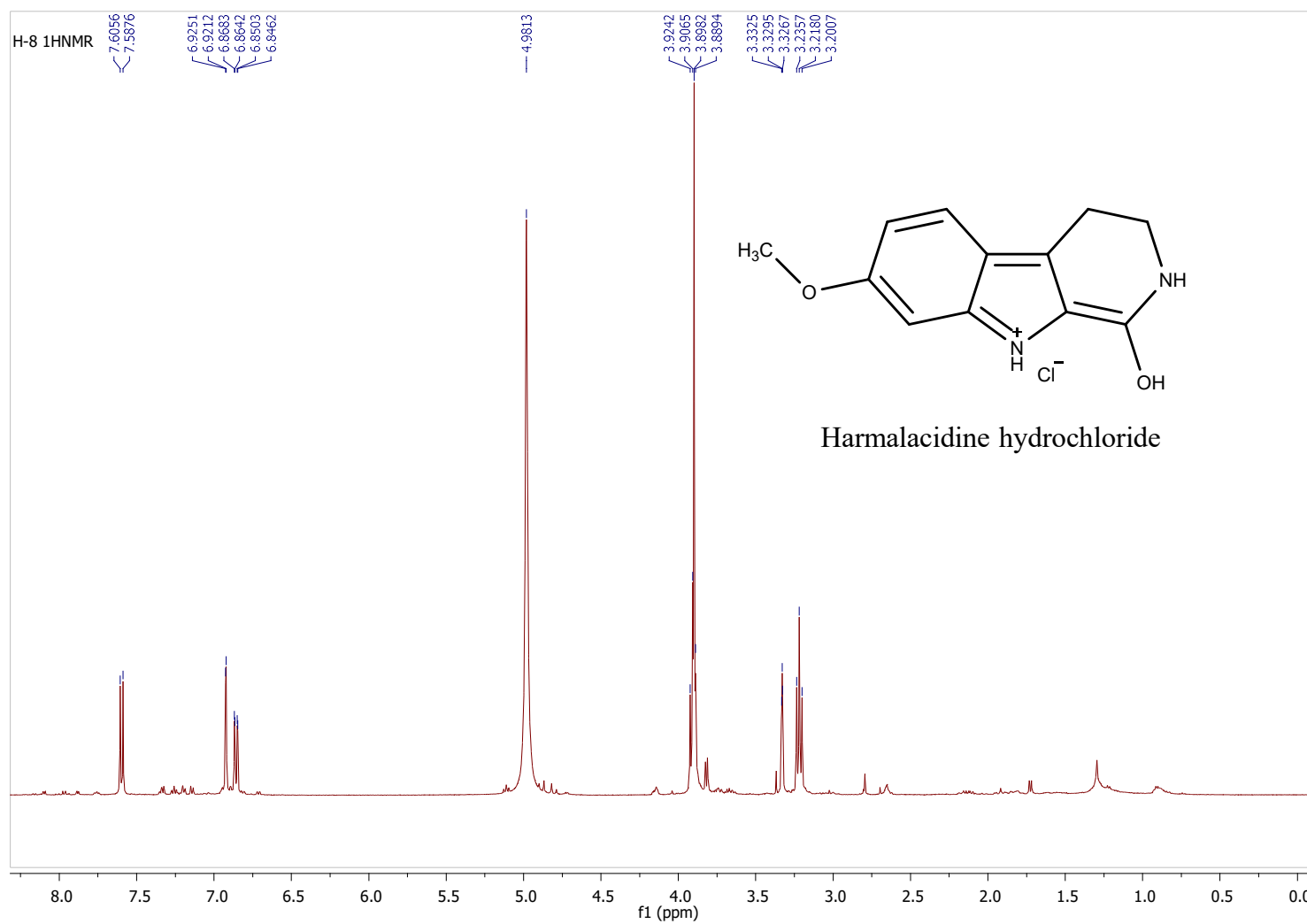

**S1 Fig.**  $^1\text{H}$  NMR spectrum of harmalacidine hydrochloride ( $\text{CD}_3\text{OD}$ , 500 MHz).

Supplement: S1 Fig — (PDF) [file pone.0335014.s003.pdf]

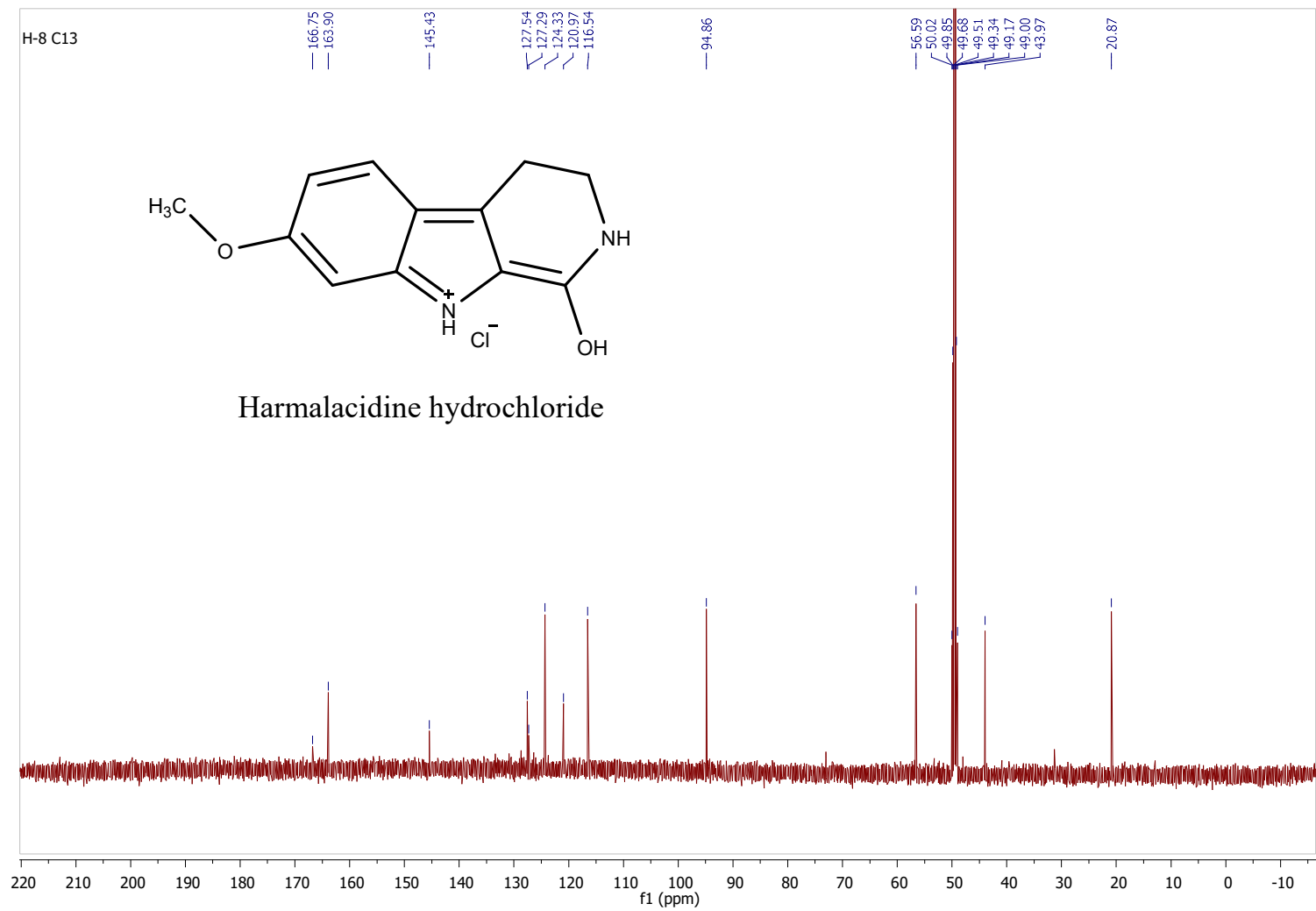

**S2 Fig.**  $^{13}\text{C}$  NMR spectrum of harmalacidine hydrochloride ( $\text{CD}_3\text{OD}$ , 125 MHz).

Supplement: S2 Fig — (PDF) [file pone.0335014.s004.pdf]

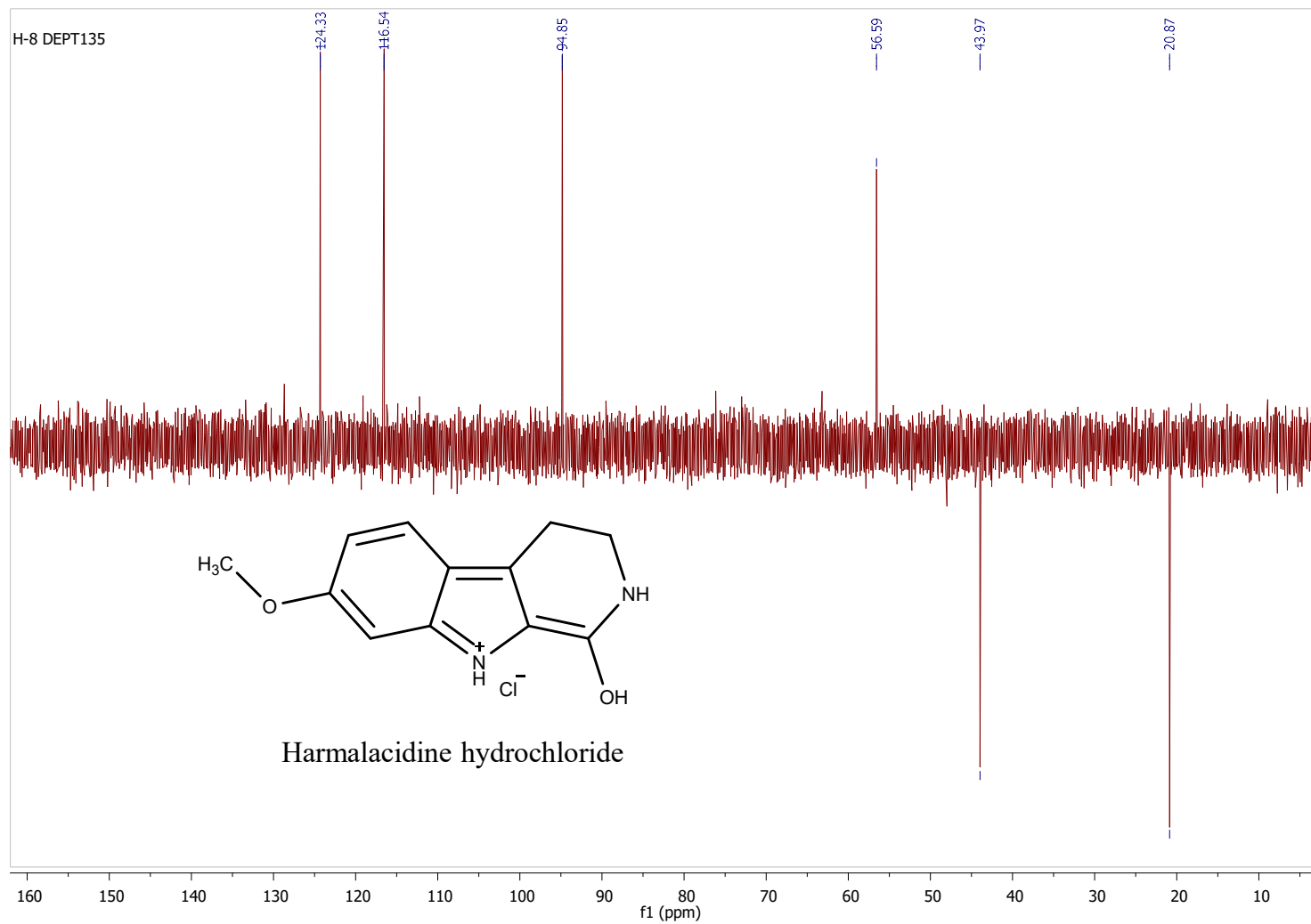

**S3 Fig.** DEPT135 spectrum of harmalacidine hydrochloride (CD<sub>3</sub>OD, 125 MHz).

Supplement: S3 Fig — (PDF) [file pone.0335014.s005.pdf]

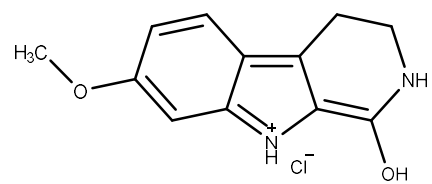

Harmalacidine hydrochloride

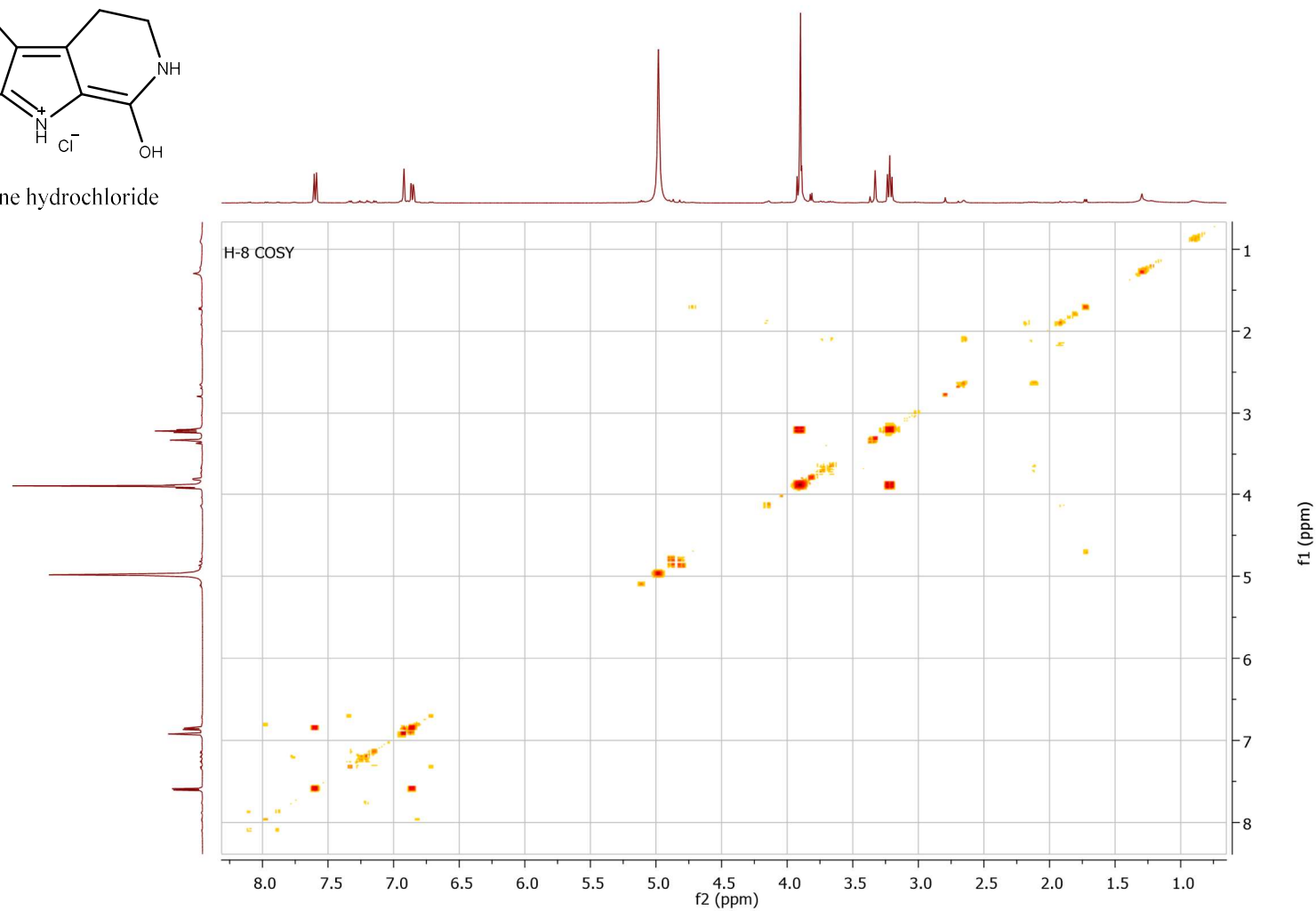

**S4 Fig.** COSY spectrum of harmalacidine hydrochloride.

Supplement: S4 Fig — (PDF) [file pone.0335014.s006.pdf]

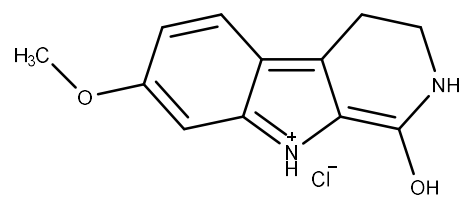

Harmalacidine hydrochloride

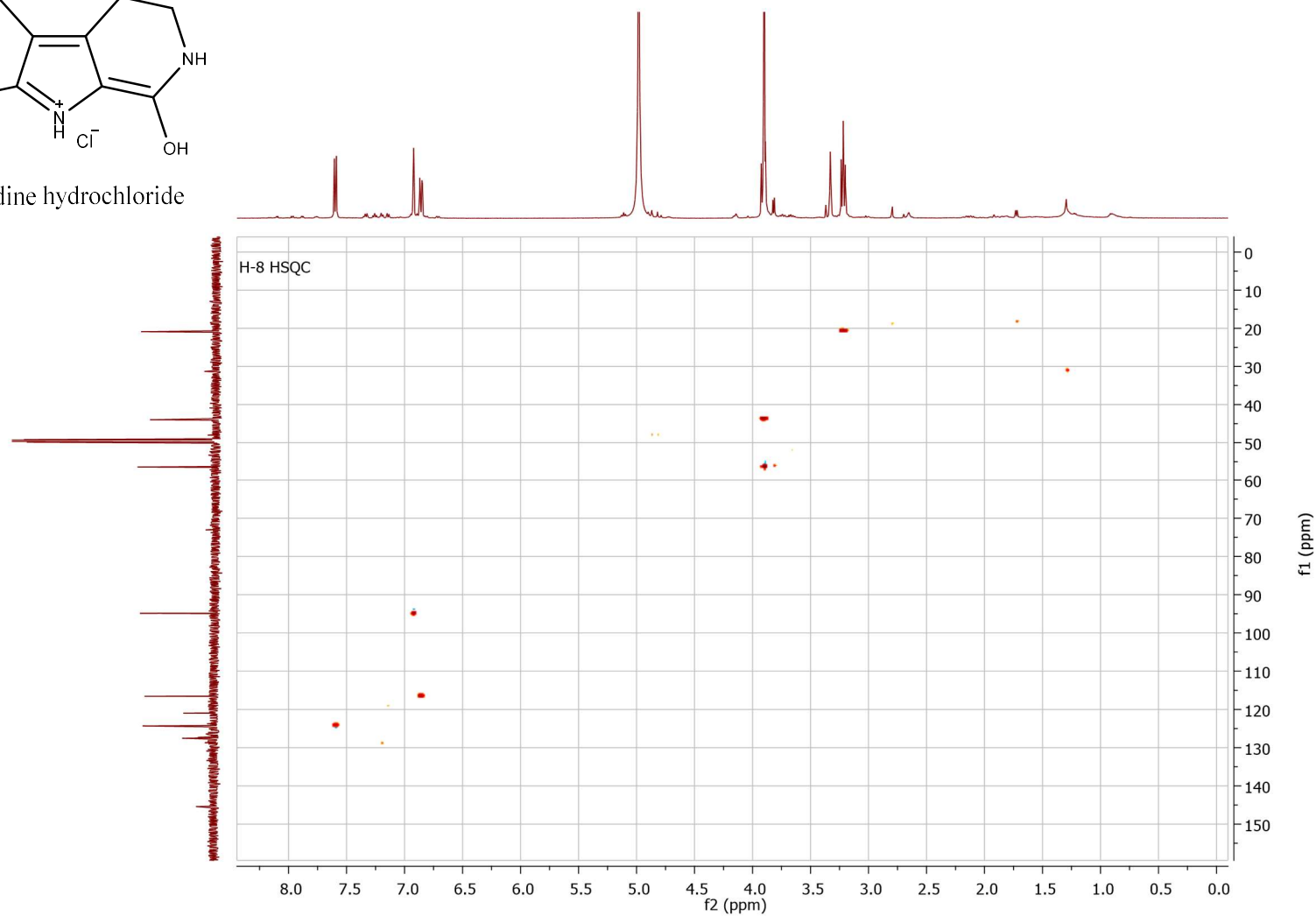

**S5 Fig.** HSQC spectrum of harmalacidine hydrochloride.

Supplement: S5 Fig — (PDF) [file pone.0335014.s007.pdf]

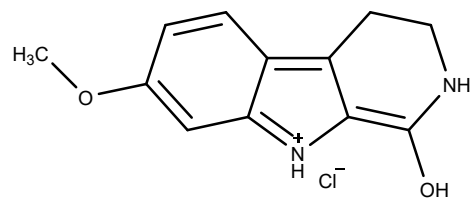

Harmalacidine hydrochloride

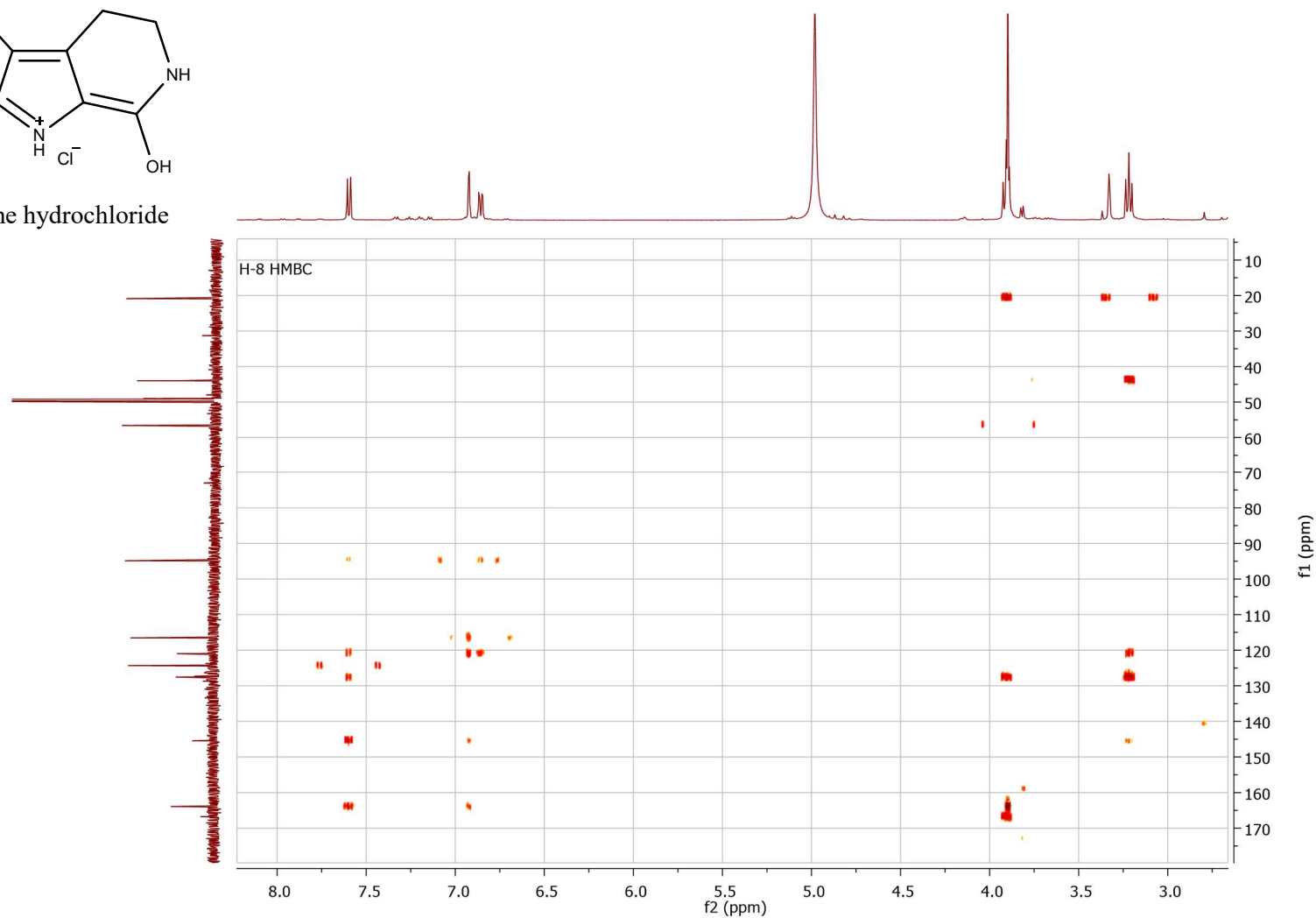

S6 Fig. HMBC spectrum of harmalacidine hydrochloride.

Supplement: S6 Fig — (PDF) [file pone.0335014.s008.pdf]
