# Supplementary material for: Antibacterial and antiviral potential of harmalacidine hydrochloride, a β-carboline alkaloid, against respiratory tract pathogens: Staphylococcus aureus and H1N1 influenza virus
Source: PLoS One. 2025 Nov 4;20(11):e0335014. doi: 10.1371/journal.pone.0335014 (PMC12585031; doi:10.1371/journal.pone.0335014)
Supplement: S1 Raw Data — (PDF) [file pone.0335014.s009.pdf]

		X	Group A			Group B		
		X Title	Data Set-A			Data Set-B		
		X	A:Y1	A:Y2	A:Y3	B:Y1	B:Y2	B:Y3
1	Title	1000.00000	0.279	0.264	0.264			
2	Title	500.00000	0.303	0.294	0.299			
3	Title	250.00000	0.313	0.317	0.309			
4	Title	125.00000	0.336	0.366	0.361			
5	Title	62.50000	0.383	0.396	0.373			
6	Title	31.25000	0.473	0.439	0.411			
7	Title	15.62500	1.493	1.117	1.305			
8	Title	7.81250	1.948	1.798	1.589			
9	Title	3.90625	2.158	2.167	2.105			
10	Title	1.00000	2.186	2.675	2.179			
11	Title		0.080	0.081	0.081			

Table format: **XY**		X	Group A			Group B		
		X Title	Data Set-A			Data Set-B		
		X	A:Y1	A:Y2	A:Y3	B:Y1	B:Y2	B:Y3
1	Title	1000.00000	0.177	0.158	0.167			
2	Title	500.00000	0.183	0.197	0.178			
3	Title	250.00000	1.073	0.889	0.981			
4	Title	125.00000	1.647	1.520	1.365			
5	Title	62.50000	1.805	1.889	1.774			
6	Title	31.25000	2.042	2.035	2.255			
7	Title	15.62500	2.445	2.346	2.333			
8	Title	7.81250	2.552	2.546	2.500			
9	Title	3.90625	2.660	2.590	2.660			
10	Title	1.00000	3.001	2.948	2.744			
11	Title		0.072	0.072	0.073			

Table format: **XY**		X	Group A			Group B		
		X Title	Data Set-A			Data Set-B		
		X	A:Y1	A:Y2	A:Y3	B:Y1	B:Y2	B:Y3
1	Title	1000.00000	0.196	0.191	0.206			
2	Title	500.00000	0.354	0.221	0.223			
3	Title	250.00000	1.061	1.002	1.121			
4	Title	125.00000	1.397	1.181	1.289			
5	Title	62.50000	1.804	1.751	1.747			
6	Title	31.25000	2.130	2.017	2.073			
7	Title	15.62500	2.172	2.146	2.131			
8	Title	7.81250	2.173	2.224	2.172			
9	Title	3.90625	2.248	2.323	2.264			
10	Title	1.00000	2.424	2.538	2.366			
11	Title		0.096	0.098	0.098			

Transform		X	A		
		X	A:1	A:2	A:3
1		3.000	0.279	0.264	0.264
2		2.699	0.303	0.294	0.299
3		2.398	0.313	0.317	0.309
4		2.097	0.336	0.366	0.361
5		1.796	0.383	0.396	0.373
6		1.495	0.473	0.439	0.411
7		1.194	1.493	1.117	1.305
8		0.893	1.948	1.798	1.589
9		0.592	2.158	2.167	2.105
10		0.000	2.186	2.675	2.179
11			0.080	0.081	0.081

Normalize		X	A		
		X	A:1	A:2	A:3
1		3.000	8.753	8.091	8.091
2		2.699	9.812	9.415	9.635
3		2.398	10.253	10.430	10.076
4		2.097	11.268	12.592	12.371
5		1.796	13.342	13.916	12.901
6		1.495	17.314	15.813	14.578
7		1.194	62.327	45.734	54.031
8		0.893	82.407	75.787	66.564
9		0.592	91.674	92.071	89.335
10		0.000	92.910	114.490	92.601
11			-0.029	0.015	0.015

		A
1	**log(in**	
2	**Best-f**	
3	Log	1.197
4	HillS	-1.550
5	IC5	15.74
6	**Std. E**	
7	Log	0.03348
8	HillS	0.1687
9	**95% C**	
10	Log	1.128 to
11	HillS	-1.896 to
12	IC5	13.44 to
13	**Good**	
14	Deg	28
15	R s	0.9516
16	Sur	1843
17	Sy.x	8.114
18		
19	**Numb**	
20	# of	30
21	# Y	30

Transform		X	A		
		X	A:1	A:2	A:3
1		3.000	0.177	0.158	0.167
2		2.699	0.183	0.197	0.178
3		2.398	1.073	0.889	0.981
4		2.097	1.647	1.520	1.365
5		1.796	1.805	1.889	1.774
6		1.495	2.042	2.035	2.255
7		1.194	2.445	2.346	2.333
8		0.893	2.552	2.546	2.500
9		0.592	2.660	2.590	2.660
10		0.000	3.001	2.948	2.744
11			0.072	0.072	0.073

Normalize		X	A		
		X	A:1	A:2	A:3
1		3.000	3.705	3.032	3.351
2		2.699	3.917	4.412	3.740
3		2.398	35.418	28.905	32.161
4		2.097	55.734	51.239	45.753
5		1.796	61.326	64.299	60.229
6		1.495	69.714	69.467	77.253
7		1.194	83.978	80.474	80.014
8		0.893	87.765	87.553	85.925
9		0.592	91.588	89.110	91.588
10		0.000	103.657	101.782	94.561
11			-0.012	-0.012	0.024

		A
1	**log(in**	
2	**Best-f**	
3	Log	1.967
4	HillS	-0.9715
5	IC5	92.64
6	**Std. E**	
7	Log	0.03413
8	HillS	0.06932
9	**95% C**	
10	Log	1.897 to [illegible]
11	HillS	-1.113 to
12	IC5	78.87 to [illegible]
13	**Good**	
14	Deg	28
15	R s	0.9642
16	Sur	1194
17	Sy.x	6.530
18		
19	**Numb**	
20	# of	30
21	# Y	30

Transform		X	A		
		X	A:1	A:2	A:3
1		3.000	0.196	0.191	0.206
2		2.699	0.354	0.221	0.223
3		2.398	1.061	1.002	1.121
4		2.097	1.397	1.181	1.289
5		1.796	1.804	1.751	1.747
6		1.495	2.130	2.017	2.073
7		1.194	2.172	2.146	2.131
8		0.893	2.173	2.224	2.172
9		0.592	2.248	2.323	2.264
10		0.000	2.424	2.538	2.366
11			0.096	0.098	0.098

Normalize		X	A		
		X	A:1	A:2	A:3
1		3.000	4.207	3.994	4.633
2		2.699	10.944	5.273	5.358
3		2.398	41.089	38.573	43.647
4		2.097	55.415	46.205	50.810
5		1.796	72.769	70.509	70.338
6		1.495	86.669	81.850	84.238
7		1.194	88.459	87.351	86.711
8		0.893	88.502	90.677	88.459
9		0.592	91.700	94.898	92.382
10		0.000	99.204	104.065	96.731
11			-0.057	0.028	0.028

		A
1	**log(in**	
2	**Best-f**	
3	Log	2.109
4	HillS	-1.149
5	IC5	128.5
6	**Std. E**	
7	Log	0.02938
8	HillS	0.08213
9	**95% C**	
10	Log	2.049 to [illegible]
11	HillS	-1.318 to
12	IC5	111.8 to 1
13	**Good**	
14	Deg	28
15	R s	0.9690
16	Sur	1051
17	Sy.x	6.127
18		
19	**Numb**	
20	# of	30
21	# Y	30

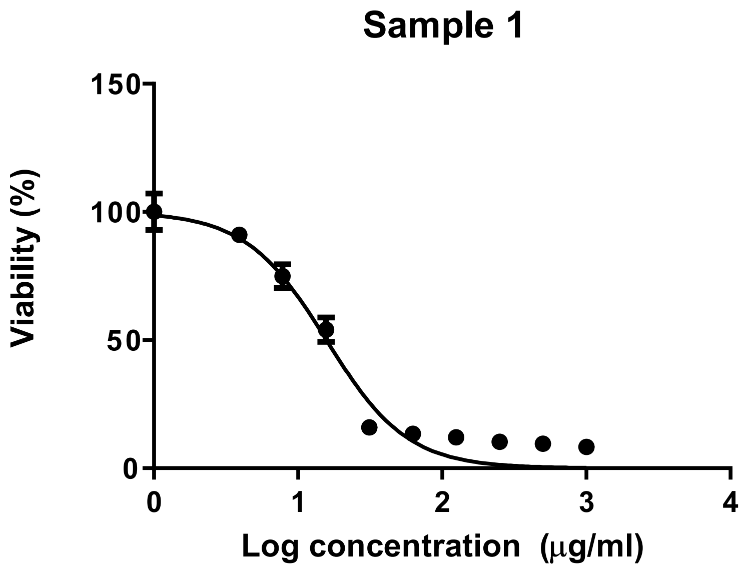
Sample 1
Viability (%)
150
100
50
0
0
1
2
3
4
Log concentration (μg/ml)

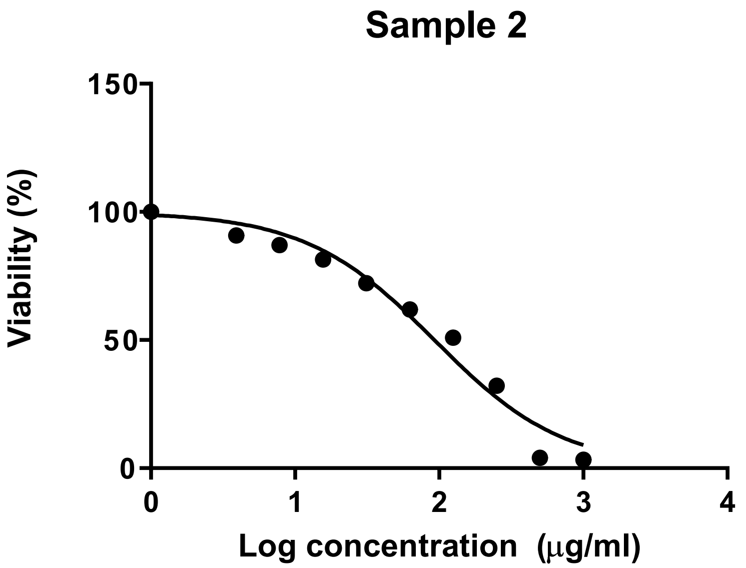
Sample 2
Viability (%)
150
100
50
0
0
1
2
3
4
Log concentration (μg/ml)

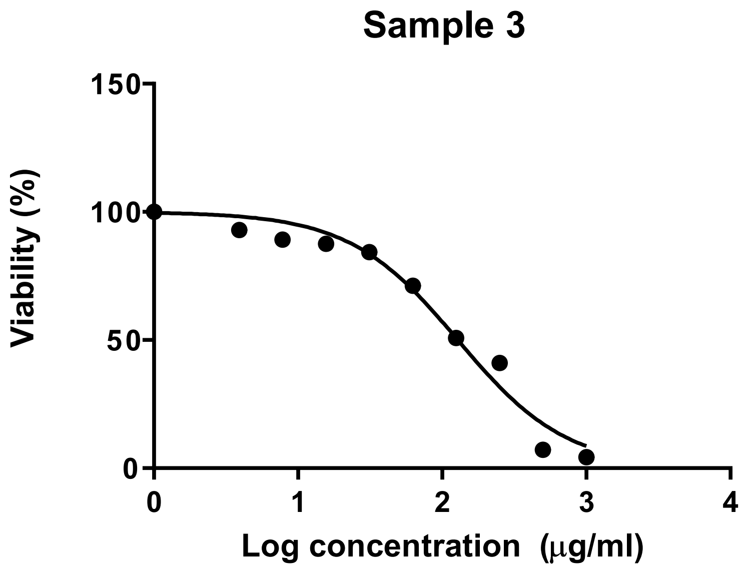
Sample 3
Viability (%)
150
100
50
0
0
1
2
3
4
Log concentration (μg/ml)

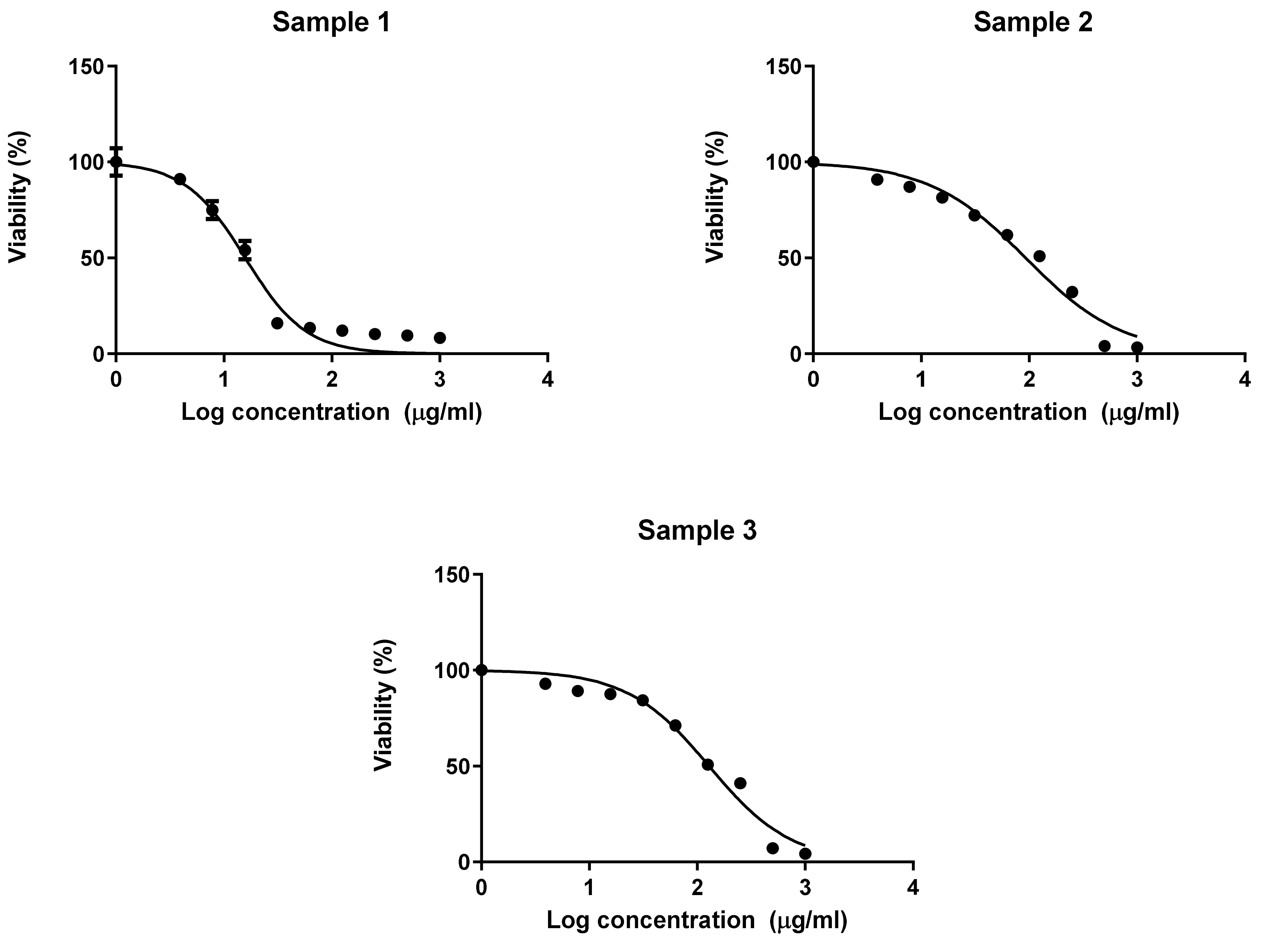
Sample 1
Viability (%)
150
100
50
0
0
1
2
3
4
Log concentration (μg/ml)
Sample 2
Viability (%)
150
100
50
0
0
1
2
3
4
Log concentration (μg/ml)
Sample 3
Viability (%)
150
100
50
0
0
1
2
3
4
Log concentration (μg/ml)
