# Supplementary material for: Antibacterial and antiviral potential of harmalacidine hydrochloride, a β-carboline alkaloid, against respiratory tract pathogens: Staphylococcus aureus and H1N1 influenza virus
Source: PLoS One. 2025 Nov 4;20(11):e0335014. doi: 10.1371/journal.pone.0335014 (PMC12585031; doi:10.1371/journal.pone.0335014)
Supplement: S2 Raw Data — (PDF) [file pone.0335014.s010.pdf]

		X	Group A		
		Conc	Log OD		
		X	A:Y1	A:Y2	A:Y3
1	Titl	0.000	0.000	0.000	0.000
2	Titl	1.875	1.400	1.700	1.800
3	Titl	3.750	5.000	5.200	5.100
4	Titl	7.500	9.200	9.000	9.300
5	Titl	15.000	30.000	30.200	30.300

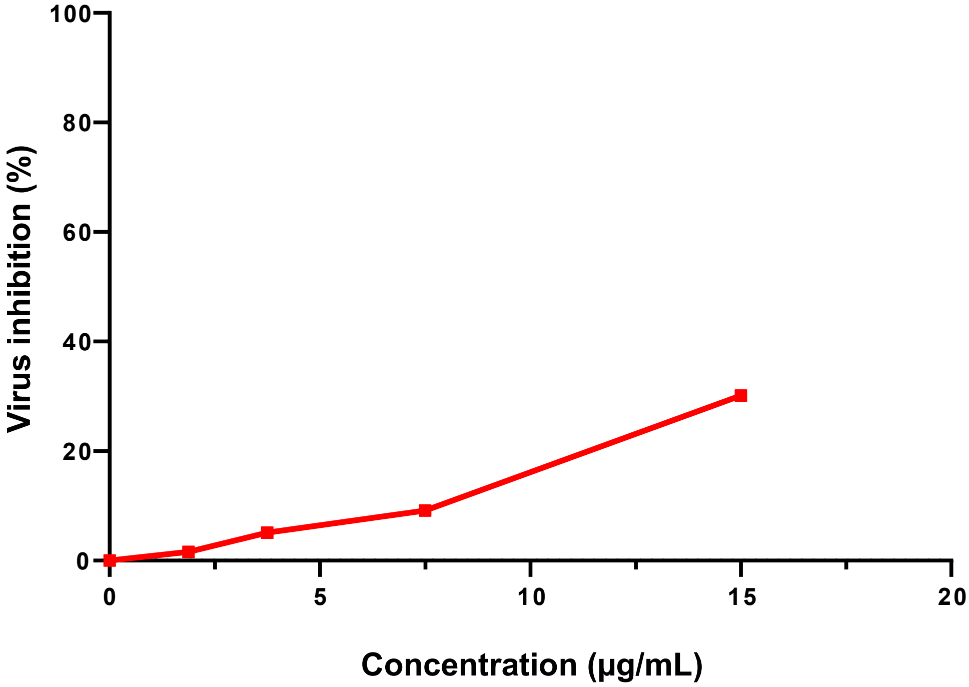

Virus inhibition (%)
100
80
60
40
20
0
0
5
10
15
20
Concentration (μg/mL)

Constant	Value
Experiment D	19//2021
Experiment ID	
Notebook ID	
Project	
Experimenter	
Protocol	
