# Supplementary material for: Antibacterial and antiviral potential of harmalacidine hydrochloride, a β-carboline alkaloid, against respiratory tract pathogens: Staphylococcus aureus and H1N1 influenza virus
Source: PLoS One. 2025 Nov 4;20(11):e0335014. doi: 10.1371/journal.pone.0335014 (PMC12585031; doi:10.1371/journal.pone.0335014)
Supplement: S3 Raw Data — (PDF) [file pone.0335014.s011.pdf]

		X	Group A		
		Conc	Log OD		
		X	A:Y1	A:Y2	A:Y3
1	Titl	0.000	0.000	0.000	0.000
2	Titl	12.500	10.000	11.000	11.200
3	Titl	25.000	13.000	13.200	14.000
4	Titl	50.000	22.200	22.000	23.000
5	Titl	100.000	60.400	60.300	60.200

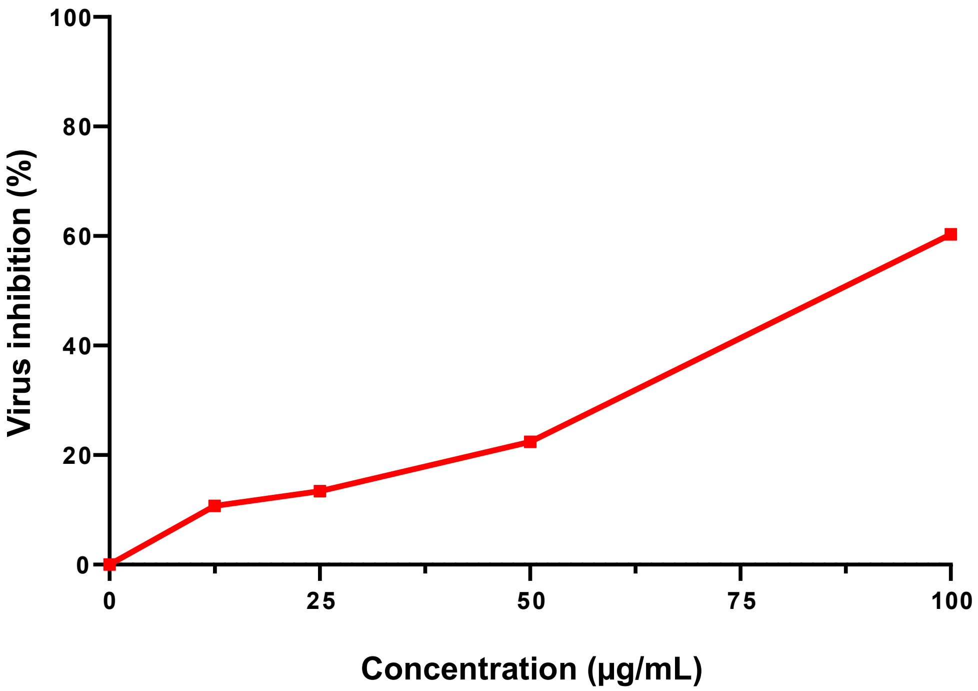
Virus inhibition (%)
100
80
60
40
20
0
0
25
50
75
100
Concentration (µg/mL)

Constant	Value
Experiment D	19//2021
Experiment ID	
Notebook ID	
Project	
Experimenter	
Protocol	
