# Supplementary material for: Antibacterial and antiviral potential of harmalacidine hydrochloride, a β-carboline alkaloid, against respiratory tract pathogens: Staphylococcus aureus and H1N1 influenza virus
Source: PLoS One. 2025 Nov 4;20(11):e0335014. doi: 10.1371/journal.pone.0335014 (PMC12585031; doi:10.1371/journal.pone.0335014)
Supplement: S4 Raw Data — (PDF) [file pone.0335014.s012.pdf]

		X	Group A			Group B			Group C		
		Time (hrs)	Log OD			Title			Data Set-C		
		X	A:Y1	A:Y2	A:Y3	B:Y1	B:Y2	B:Y3	C:Y1	C:Y2	C:Y3
1	Titl	0.0	0.000	0.000	0.000				0.000	0.000	0.000
2	Titl	1.0	0.050	0.051	0.052				0.091	0.090	0.092
3	Titl	2.0	0.057	0.058	0.059				0.120	0.123	0.126
4	Titl	4.0	0.061	0.060	0.062				0.138	0.130	0.135

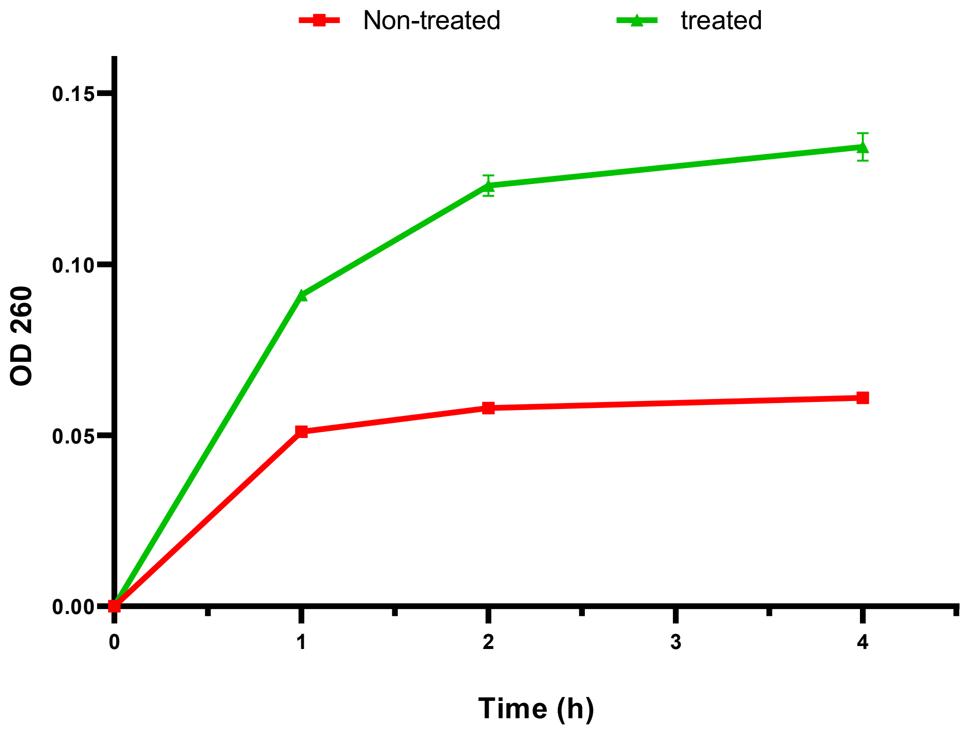
Non-treated
treated
0.15
0.10
0.05
0.00
OD 260
0
1
2
3
4
Time (h)

Constant	Value
Experiment D	19//2021
Experiment ID	
Notebook ID	
Project	
Experimenter	
Protocol	
