# Supplementary material for: Antibacterial and antiviral potential of harmalacidine hydrochloride, a β-carboline alkaloid, against respiratory tract pathogens: Staphylococcus aureus and H1N1 influenza virus
Source: PLoS One. 2025 Nov 4;20(11):e0335014. doi: 10.1371/journal.pone.0335014 (PMC12585031; doi:10.1371/journal.pone.0335014)
Supplement: S5 Raw Data — (PDF) [file pone.0335014.s013.pdf]

		X	Group A			Group B			Group C		
		Time (hrs)	Log OD			Title			Data Set-C		
		X	A:Y1	A:Y2	A:Y3	B:Y1	B:Y2	B:Y3	C:Y1	C:Y2	C:Y3
1	Title	0.0	0.000	0.000	0.000				0.000	0.000	0.000
2	Title	10.0	0.000	0.000	0.000				0.018	0.019	0.018
3	Title	20.0	0.000	0.000	0.000				0.020	0.021	0.022
4	Title	30.0	0.006	0.008	0.007				0.030	0.032	0.033
5	Title	40.0	0.009	0.009	0.008				0.035	0.036	0.037
6	Title	50.0	0.013	0.012	0.011				0.049	0.048	0.050
7	Title	60.0	0.019	0.020	0.018				0.061	0.062	0.063
8	Title	70.0	0.028	0.029	0.030				0.067	0.068	0.069

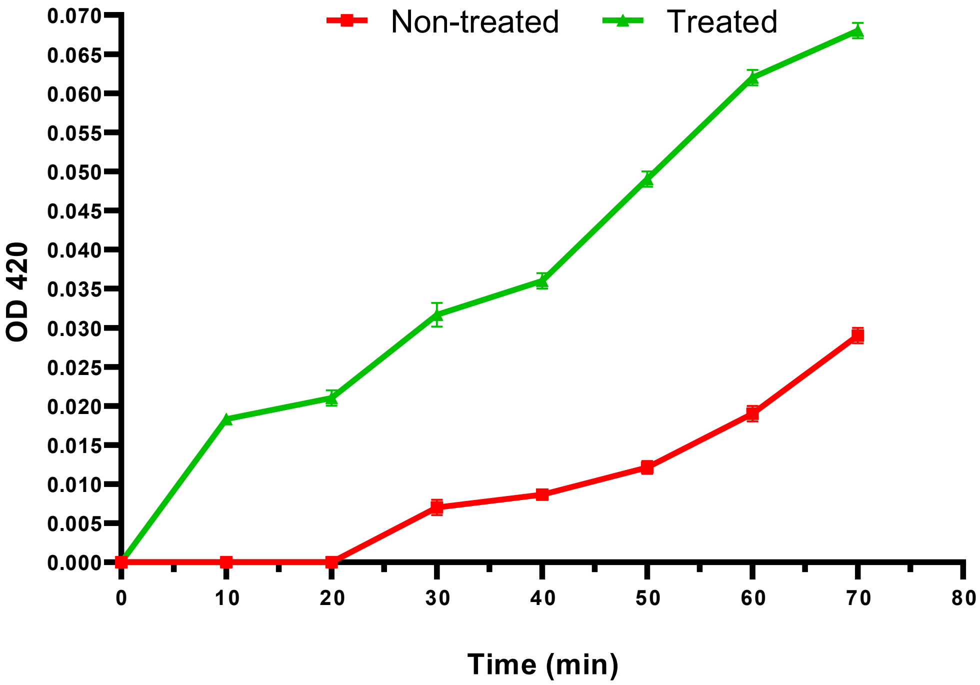

Non-treated
Treated
OD 420
0.070
0.065
0.060
0.055
0.050
0.045
0.040
0.035
0.030
0.025
0.020
0.015
0.010
0.005
0.000
0
10
20
30
40
50
60
70
80
Time (min)

Constant	Value
Experiment D	19//2021
Experiment ID	
Notebook ID	
Project	
Experimenter	
Protocol	
