# Supplementary material for: Antibacterial and antiviral potential of harmalacidine hydrochloride, a β-carboline alkaloid, against respiratory tract pathogens: Staphylococcus aureus and H1N1 influenza virus
Source: PLoS One. 2025 Nov 4;20(11):e0335014. doi: 10.1371/journal.pone.0335014 (PMC12585031; doi:10.1371/journal.pone.0335014)
Supplement: S6 Raw Data — (PDF) [file pone.0335014.s014.pdf]

Table format: **Column**		Group A	Group B	Group C	Group D	Group E
		C2	C5	C6	C8	C10
1	Title	0.110	0.230	0.400	0.21	0.41
2	Title	0.150	0.240	0.410	0.23	0.40
3	Title	0.170	0.250	0.420	0.21	0.42

Table format: **Grouped**		Group A		Group B		Group C	
		Data Set-A		Data Set-B		Data Set-C	
		Mean	SD	Mean	SD	Mean	SD
1	S2	0.40	0.02	0.40	0.04	0.30	0.02
2	S4	0.30	0.01	0.30	0.03	0.19	0.04
3	S5	0.50	0.00	0.32	0.05	0.16	0.06
4	S7	0.23	0.03	0.31	0.03	0.30	0.04
5	S10	0.16	0.03	0.20	0.05	0.40	0.03
6	S11	0.19	0.03	0.44	0.05	0.30	0.06
7	S13	0.45	0.02	0.31	0.03	0.34	0.05

**Ordinary one-way ANOVA**<br>ANOVA results						
1	Table Analyzed	Data 1				
2	Data sets analyzed	A-E				
3						
4	**ANOVA summary**					
5	F	159.2				
6	P value	<0.0001				
7	P value summary	****				
8	Significant diff. among means (P < 0.05)?	Yes				
9	R squared	0.9845				
10						
11	**Brown-Forsythe test**					
12	F (DFn, DFd)	0.8421 (4, 10)				
13	P value	0.5293				
14	P value summary	ns				
15	Are SDs significantly different (P < 0.05)?	No				
16						
17	**Bartlett's test**					
18	Bartlett's statistic (corrected)					
19	P value					
20	P value summary					
21	Are SDs significantly different (P < 0.05)?					
22						
23	**ANOVA table**	**SS**	**DF**	**MS**	**F (DFn, DFd)**	**P value**
24	Treatment (between columns)	0.1740	4	0.04351	F (4, 10) = 159.2	P<0.0001
25	Residual (within columns)	0.002733	10	0.0002733		
26	Total	0.1768	14			
27						
28	**Data summary**					
29	Number of treatments (columns)	5				
30	Number of values (total)	15				

**Ordinary one-way ANOVA** Multiple comparisons					
1	Number of families	1			
2	Number of comparisons per family	10			
3	Alpha	0.05			
4					
5	**Tukey's multiple comparisons test**	**Mean Diff.**	**95.00% CI of diff.**	**Below threshold?**	**Summary**
6	C2 vs. C5	-0.09667	-0.1411 to -0.05224	Yes	***
7	C2 vs. C6	-0.2667	-0.3111 to -0.2222	Yes	****
8	C2 vs. C8	-0.07333	-0.1178 to -0.02891	Yes	**
9	C2 vs. C10	-0.2667	-0.3111 to -0.2222	Yes	****
10	C5 vs. C6	-0.1700	-0.2144 to -0.1256	Yes	****
11	C5 vs. C8	0.02333	-0.02109 to 0.06776	No	ns
12	C5 vs. C10	-0.1700	-0.2144 to -0.1256	Yes	****
13	C6 vs. C8	0.1933	0.1489 to 0.2378	Yes	****
14	C6 vs. C10	0.000	-0.04443 to 0.04443	No	ns
15	C8 vs. C10	-0.1933	-0.2378 to -0.1489	Yes	****
16					
17	**Test details**	**Mean 1**	**Mean 2**	**Mean Diff.**	**SE of diff.**
18	C2 vs. C5	0.1433	0.2400	-0.09667	0.01350
19	C2 vs. C6	0.1433	0.4100	-0.2667	0.01350
20	C2 vs. C8	0.1433	0.2167	-0.07333	0.01350
21	C2 vs. C10	0.1433	0.4100	-0.2667	0.01350
22	C5 vs. C6	0.2400	0.4100	-0.1700	0.01350
23	C5 vs. C8	0.2400	0.2167	0.02333	0.01350
24	C5 vs. C10	0.2400	0.4100	-0.1700	0.01350
25	C6 vs. C8	0.4100	0.2167	0.1933	0.01350
26	C6 vs. C10	0.4100	0.4100	0.000	0.01350
27	C8 vs. C10	0.2167	0.4100	-0.1933	0.01350

1				
2				
3				
4				
5	**Adjusted P Value**			
6	0.0002	A-B		
7	<0.0001	A-C		
8	0.0021	A-D		
9	<0.0001	A-E		
10	<0.0001	B-C		
11	0.4601	B-D		
12	<0.0001	B-E		
13	<0.0001	C-D		
14	>0.9999	C-E		
15	<0.0001	D-E		
16				
17	**n1**	**n2**	**q**	**DF**
18	3	3	10.13	10
19	3	3	27.94	10
20	3	3	7.683	10
21	3	3	27.94	10
22	3	3	17.81	10
23	3	3	2.445	10
24	3	3	17.81	10
25	3	3	20.25	10
26	3	3	0.000	10
27	3	3	20.25	10

**Ordinary one-way ANOVA**<br>ANOVA results						
1	Table Analyzed	Data 1				
2	Data sets analyzed	A-E				
3						
4	**ANOVA summary**					
5	F	159.2				
6	P value	<0.0001				
7	P value summary	****				
8	Significant diff. among means (P < 0.05)?	Yes				
9	R squared	0.9845				
10						
11	**Brown-Forsythe test**					
12	F (DFn, DFd)	0.8421 (4, 10)				
13	P value	0.5293				
14	P value summary	ns				
15	Are SDs significantly different (P < 0.05)?	No				
16						
17	**Bartlett's test**					
18	Bartlett's statistic (corrected)					
19	P value					
20	P value summary					
21	Are SDs significantly different (P < 0.05)?					
22						
23	**ANOVA table**	**SS**	**DF**	**MS**	**F (DFn, DFd)**	**P value**
24	Treatment (between columns)	0.1740	4	0.04351	F (4, 10) = 159.2	P<0.0001
25	Residual (within columns)	0.002733	10	0.0002733		
26	Total	0.1768	14			
27						
28	**Data summary**					
29	Number of treatments (columns)	5				
30	Number of values (total)	15				

**Ordinary one-way ANOVA**<br>Multiple comparisons					
1	Number of families	1			
2	Number of comparisons per family	10			
3	Alpha	0.05			
4					
5	**Tukey's multiple comparisons test**	**Mean Diff.**	**95.00% CI of diff.**	**Below threshold?**	**Summary**
6	C2 vs. C5	-0.09667	-0.1411 to -0.05224	Yes	***
7	C2 vs. C6	-0.2667	-0.3111 to -0.2222	Yes	****
8	C2 vs. C8	-0.07333	-0.1178 to -0.02891	Yes	**
9	C2 vs. C10	-0.2667	-0.3111 to -0.2222	Yes	****
10	C5 vs. C6	-0.1700	-0.2144 to -0.1256	Yes	****
11	C5 vs. C8	0.02333	-0.02109 to 0.06776	No	ns
12	C5 vs. C10	-0.1700	-0.2144 to -0.1256	Yes	****
13	C6 vs. C8	0.1933	0.1489 to 0.2378	Yes	****
14	C6 vs. C10	0.000	-0.04443 to 0.04443	No	ns
15	C8 vs. C10	-0.1933	-0.2378 to -0.1489	Yes	****
16					
17	**Test details**	**Mean 1**	**Mean 2**	**Mean Diff.**	**SE of diff.**
18	C2 vs. C5	0.1433	0.2400	-0.09667	0.01350
19	C2 vs. C6	0.1433	0.4100	-0.2667	0.01350
20	C2 vs. C8	0.1433	0.2167	-0.07333	0.01350
21	C2 vs. C10	0.1433	0.4100	-0.2667	0.01350
22	C5 vs. C6	0.2400	0.4100	-0.1700	0.01350
23	C5 vs. C8	0.2400	0.2167	0.02333	0.01350
24	C5 vs. C10	0.2400	0.4100	-0.1700	0.01350
25	C6 vs. C8	0.4100	0.2167	0.1933	0.01350
26	C6 vs. C10	0.4100	0.4100	0.000	0.01350
27	C8 vs. C10	0.2167	0.4100	-0.1933	0.01350

1				
2				
3				
4				
5	**Adjusted P Value**			
6	0.0002	A-B		
7	<0.0001	A-C		
8	0.0021	A-D		
9	<0.0001	A-E		
10	<0.0001	B-C		
11	0.4601	B-D		
12	<0.0001	B-E		
13	<0.0001	C-D		
14	>0.9999	C-E		
15	<0.0001	D-E		
16				
17	**n1**	**n2**	**q**	**DF**
18	3	3	10.13	10
19	3	3	27.94	10
20	3	3	7.683	10
21	3	3	27.94	10
22	3	3	17.81	10
23	3	3	2.445	10
24	3	3	17.81	10
25	3	3	20.25	10
26	3	3	0.000	10
27	3	3	20.25	10

Paired t test		
1	Table Analyzed	1
2		
3	Column B	Data Set-B
4	vs.	vs.
5	Column A	Data Set-A

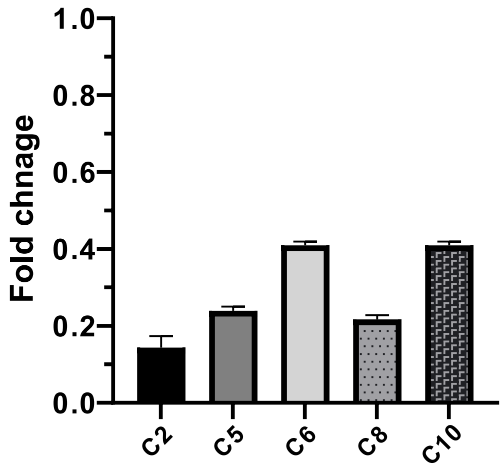

Fold chnage
1.0
0.8
0.6
0.4
0.2
0.0
C2
C5
C6
C8
C10

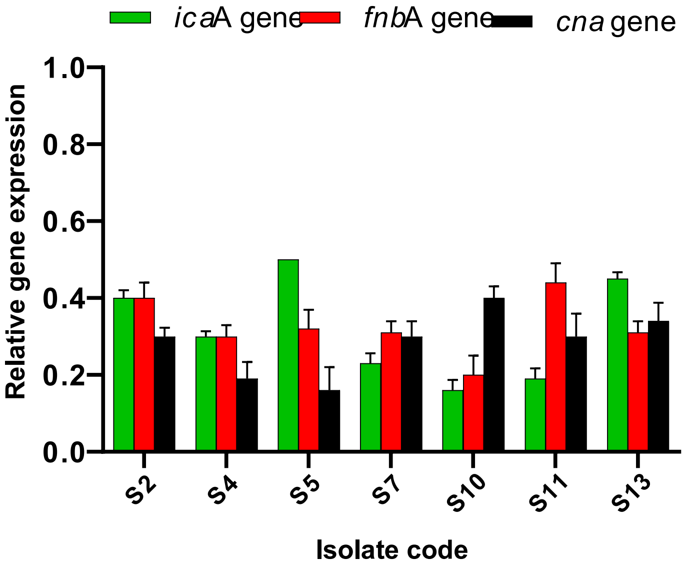

*ica*A gene
*fnb*A gene
cna gene
Relative gene expression
1.0
0.8
0.6
0.4
0.2
0.0
S2
S4
S5
S7
S10
S11
S13
Isolate code

Constant	Value
Experiment D	29//2022
Experiment ID	
Notebook ID	
Project	
Experimenter	
Protocol	
