# Supplementary material for: Antibacterial and antiviral potential of harmalacidine hydrochloride, a β-carboline alkaloid, against respiratory tract pathogens: Staphylococcus aureus and H1N1 influenza virus
Source: PLoS One. 2025 Nov 4;20(11):e0335014. doi: 10.1371/journal.pone.0335014 (PMC12585031; doi:10.1371/journal.pone.0335014)
Supplement: S1 Method — (PDF) [file pone.0335014.s015.pdf]

S1 Method. Molecular docking study

The molecular docking study was executed using the new version of Autodock vina 1.2.6 [1, 2]. The crystal structures for the target protein receptors were retrieved from the RCSB protein data bank in PDB format, with their corresponding PDB codes listed in **Table S3**. The investigated compounds and the used proteins crystal structures were prepared for the docking study using Autodock tools. A grid box with the dimensions listed in **Table S3** and a spacing of 0.375 Å was used to define the binding sites in all docking studies. Each grid box was centered on the co-crystallized ligand associated with its respective receptor or covering the whole protein structure in case of blind docking. The specific grid coordinates for each receptor are provided in **Table S3**. The docking protocol was validated by removing the co-crystallized ligand from each receptor and re-docking it into the active site using AutoDock Vina in case of H1N1 neuraminidase and polymerase basic protein 2 (PB2) enzymes. The root mean square deviation (RMSD) between the re-docked and original co-crystallized ligand was then calculated, yielding values below 2.0 Å, confirming the reliability of the docking procedure. The obtained docking poses with the least RMSD values were visualized in 3D and 2D styles using Biovia Discovery Studio [3].

References

1. Trott O, Olson AJ. AutoDock Vina: improving the speed and accuracy of docking with a new scoring function, efficient optimization, and multithreading. J comput chem. 2010;31(2):455-61. https://doi.org/10.1002/jcc.21334. PMID: 19499576
2. Eberhardt J, Santos-Martins D, Tillack AF, Forli S. AutoDock Vina 1.2. 0: New docking methods, expanded force field, and python bindings. J Chem Inf Model. 2021;61(8):3891-8. https://doi.org/10.1021/acs.jcim.1c00203. PMID: 34278794
3. Baroroh U BM, Muscifa ZS, Destiarani W, Rohmatullah FG, Yusuf M. . Molecular interaction analysis and visualization of protein-ligand docking using Biovia Discovery Studio Visualizer. Indo J Comput Biol. 2023;2(1):22-30. https://doi.org/10.24198/ijcb.v2i1.46322.
